# Hybrid neural–cognitive models reveal how memory shapes human reward learning

**DOI:** 10.1038/s41562-025-02324-0

**Published:** 2026-02-05

**Authors:** Maria K. Eckstein, Christopher Summerfield, Nathaniel D. Daw, Kevin J. Miller

**Affiliations:** 1 Google DeepMind, London, UK; 2 Department of Experimental Psychology, University of Oxford, Oxford, UK; 3 Princeton Neuroscience Institute and Department of Psychology, Princeton University, Princeton, NJ USA; 4 Sainsbury Wellcome Centre, University College London, London, UK

**Keywords:** Human behaviour, Computational science

## Abstract

A long-standing challenge for psychology and neuroscience is to understand the transformations by which past experiences shape future behaviour. Reward-guided learning is typically modelled using simple reinforcement learning (RL) algorithms. In RL, a handful of incrementally updated internal variables both summarize past rewards and drive future choice. Here we describe work that questions the assumptions of many RL models. We adopt a hybrid modelling approach that integrates artificial neural networks into interpretable cognitive architectures, estimating a maximally general form for each algorithmic component and systematically evaluating its necessity and sufficiency. Applying this method to a large dataset of human reward-learning behaviour, we show that successful models require independent and flexible memory variables that can track rich representations of the past. Using a modelling approach that combines predictive accuracy and interpretability, these results call into question an entire class of popular RL models based on incremental updating of scalar reward predictions.

## Abstract (web summary)

Using artificial neural networks applied to human data, Eckstein et al. show that good models of reinforcement learning require memory components that track representations of the past.

## Main

Reward-guided decisions are widely assumed to depend on a small number of latent variables that concisely summarize the history of actions and rewards and are calculated using simple incremental updates after each experience. For example, within the framework of reinforcement learning (RL), standard cognitive models posit that choices are based on ‘*Q*-values’, which approximate the expected reward associated with each action and are calculated by repeatedly applying an incremental learning rule that compares the actual outcome to its previous estimate^1,2^. Such models are often simply called ‘RL models’, and they form the foundation for many studies investigating the psychology and neuroscience of reward-guided learning. These models have achieved an impressive record of success, providing computational explanations for basic as well as complex learning phenomena^3–9^ and for neural correlates of reward-guided learning in a variety of tasks and species^10–12^.

However, the literature has also accumulated a number of observations that these models do not easily account for. For example, individual events in the past can disproportionately affect behaviour^13–17^, suggesting that task-relevant memory contains more than *Q*-value-like summary statistics of the reward history. Additionally, behaviour is often sensitive to global statistics of the past (for example, the range of rewards or the grouping of choice options) that are not easily captured by standard RL models^18–21^. Lastly, neural signals previously thought to relate straightforwardly to *Q*-values have been found to show marked diversity that is in tension with standard RL models^22–26^. These findings collectively suggest that the memory representations that humans and animals use to make reward-based choices go beyond incrementally learned summary statistics and may rely on a variety of additional internal memory mechanisms. However, a coherent computational account of such a learning algorithm is lacking.

Artificial neural networks (ANNs) are able to model highly expressive functions^27^. Sequential tasks can be modelled using recurrent neural networks (RNNs), which can learn to represent the past using high-dimensional internal states; these states are derived by memory mechanisms that are implemented in a potentially large number of trainable network parameters. With the ability to learn complex, time-dependent mapping functions, RNNs seem able to capture both the long-term dependencies and the potentially complex learning mechanisms that underlie human behaviour during reward-based learning^28–31^. These networks have the advantage that they typically capture more behavioural variance than handcrafted cognitive models, providing an estimate of the model performance that is possible for a given dataset^30,32,33^. However, fitting behaviour with RNNs typically comes at the expense of interpretability—unlike in classic cognitive modes such as RL, in which each parameter serves a prescribed role, their computations typically require substantial additional work to interpret^34,35^.

**Fig. 1:**
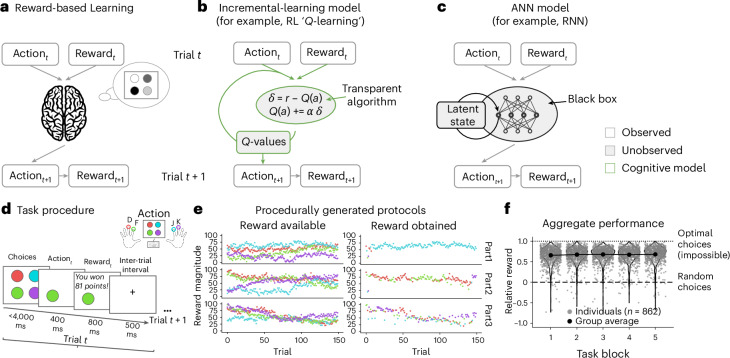
Overview. **a**–**c**, Cognitive modelling. Panel **a** illustrates reward-based learning. Reward-based learning tasks involve choosing one action at a time to win a reward, in an iterative fashion involving many trials. Panel **b** illustrates an incremental-learning model. Variants of RL, specifically *Q*-learning, are popular behavioural models for such tasks. *Q*-values approximate the expected reward associated with each action on the basis of an incremental, trial-wise delta-rule update. Panel **c** illustrates an ANN model. While classic cognitive models facilitate understanding of the underlying mechanism, ANNs typically predict empirical behaviour more accurately. **d**–**f**, Experimental design. Panel **d** shows the task procedure. On each trial, participants saw the same four stimuli, pressed a key to select one and obtained the corresponding reward (1–100 points). Each task lasted for 150 trials. Panel **e** shows examples of procedurally generated protocols. Each row shows the protocol for one of three example participants. The number of points available for each action diffused over time (left), independently for each action (colour). A different reward schedule was used for each participant and each task block. Participants’ choices (right) reflected individual reward schedules. Panel **f** shows aggregate performance. Each participant (grey dots) performed multiple task blocks (horizontal axis). ‘Relative reward’ is a measure of task performance that is comparable across different reward schedules (see ‘Behavioural analyses’ in Methods). The black dots show means over participants, and the error bars (almost invisible) indicate standard errors.

A budding research field has started to combine ANNs and classic cognitive models^28,31–33,36^. Whereas handcrafted cognitive models are interpretable but frequently underfit the data, ANNs are sufficiently expressive to model complex behaviours but usually hard to understand. For example, Peterson et al.^36^ iteratively replaced components of a classic computational model with more expressive ANN counterparts to test increasingly general theories of human decision-making, using gambling tasks. Here we extend this approach to study reward-based learning and memory, which requires modelling both how information about the past is integrated into memory and how the contents of memory are used to guide choice. To do this, we created a hybrid neural–cognitive method that flexibly interpolates between a classic RL model (Fig. 1b) and an RNN (Fig. 1c). Iteratively replacing RL model components with flexible ANNs, we measured which relaxation of constraints improved the model’s ability to capture human behaviour. We then inspected the best model’s fitted ANN modules to shed light on the underlying mechanisms and to understand how experience shapes memory representations and how these representations drive choice.

## Results

We collected a large dataset from a reward-learning task in which human participants repeatedly chose among four possible actions, which were rewarded according to noisy reward magnitudes that drifted over time (a non-stationary ‘bandit’ task; Fig. 1e)^37^. On each trial of the task, the participants selected one of the four actions and were given the corresponding reward (Fig. 1d). We collected the dataset online (880 participants, 862 of whom passed the inclusion criteria; 4,134 task blocks; 617,871 valid trials; all participants provided informed consent in accordance with Google DeepMind’s Human Behavioural Research Ethics Committee, and the study complied with all relevant ethical regulations), which is comparable in size to the largest existing datasets from related tasks^38,39^. Participants tended to choose the actions with larger rewards, indicating that they successfully learned the task (average rewards exceeded chance (*t*_861_ = 149.2; *P* < 0.001; *d* = 5.09; 95% confidence interval (CI) of relative rewards, (66.2, 67.9)) and were numerically above chance on 4,085/4,134 task blocks; Fig. 1f). Both the large size of our dataset and the variability of reward contingencies between participants were crucial to our approach because they allowed RNNs and hybrid models to extract additional variance compared with basic RL models (Supplementary Fig. 7).

**Fig. 2:**
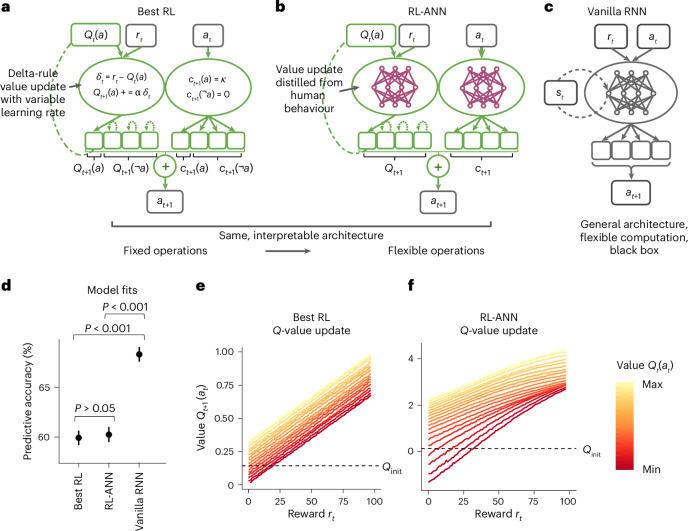
Best RL and RL-like models. **a**–**c**, Model architectures. Best RL (**a**) is the best handcrafted model based on *Q*-learning, identified using extensive model comparison (Supplementary Results and Supplementary Table 2). It contains a standard *Q*-value update (left oval) with decay of unchosen action values (left oval, dotted recurrent arrows for *Q*(¬*a*)), as well as a reward-agnostic choice perseveration mechanism (right oval). The outputs of both computations are combined additively to sample the next choice. RL-ANN (**b**) has the same architecture as Best RL, consisting of a reward module that computes *Q*-values (left oval) and an action module that computes a perseveration kernel (right oval). However, RL-ANN uses ANNs to allow each module to perform any update rule, making it a generalization of linear update models that encompasses Best RL as a special case. Vanilla RNN (**c**) is a standard RNN and the most flexible model. It provides an upper bound in terms of behavioural prediction. **d**, Model fits. Predictive accuracy was derived from the loss of each fitted model to held-out participants not seen during training (see ‘Model training’ in Methods). Best RL and RL-ANN predicted human choices significantly worse than Vanilla RNN, with no significant difference between them, according to two-sided *t*-tests (see the main text for statistics). The data are presented as mean values over held-out blocks (*n* = 413) plus/minus s.e.m. **e**,**f**, Reward processing. In classic *Q*-learning as modelled by Best RL (**e**), updated values *Q*_*t*__+1_(*a*_*t*_) increase monotonically both in the observed reward *r*_*t*_ and in the previous value *Q*_*t*_(*a*_*t*_) (colour), with strictly linear relationships (for model details and equations, see ‘Model architectures’ in Methods). After fitting to human behaviour, RL-ANN (**f**) acquired a qualitatively similar update rule with monotonically increasing and near-linear relationships. For ease of visualization, we averaged sampled values *Q*_*t*_(*a*_*t*_) (colour) within quantile groups to obtain discrete lines.

We first modelled this dataset using the two extreme approaches, a classic RL-based incremental-update model and a generic RNN. We identified the best RL model (Fig. 2a) through systematic comparison between many RL model variants, using standard methods^40,41^ (Supplementary Table 2; implementation details are provided in ‘Model architectures’ in Methods). Specifically, we started with the simplest model (called ‘Simple RL’), a tabular *Q*-learner with two free model parameters (learning rate and inverse decision temperature), and fitted it to participant behaviour by identifying the parameter values that maximized the negative log-likelihood of human behaviour under the model in the training split of the dataset. We then tested a variety of modifications to Simple RL that have been explored in the literature, including *Q*-value forgetting^4,42^ and a parallel perseveration module that learns from actions rather than rewards^7,43,44^. Among all tested RL model variants, we identified a winning model with six free parameters, called ‘Best RL’. Best RL consists of two submodules. The ‘reward module’ takes as inputs the observed reward, denoted *r*_*t*_, and the value, *Q*_*t*_(*a*_*t*_), of the action *a*_*t*_ that led to this reward, and calculates an updated *Q*-value, *Q*_*t*+1_(*a*_*t*_), for this action, using the equations specified in Fig. 2a (left). In Best RL, *Q*-values *Q*_*t*__+1_(*a*_*t*_) hence are linear in both the reward *r*_*t*_ and the previous value *Q*_*t*_(*a*_*t*_), such that larger rewards and larger previous values lead to monotonically larger updated values (Fig. 2e). Best RL’s forgetting mechanism gradually decays *Q*-values back to the initial value *Q*_init_. The reward module hence captures pure reward-based learning. In addition, Best RL has an ‘action module’, which takes as input the previous action, *a*_*t*_, and sets its perseveration indicator *c*_*t*__+1_(*a*_*t*_) to a value determined by a free parameter. This allows the model to express either action repetition (*c*_*t*_(*a*_*t*_) > 0) or action switching (*c*_*t*_(*a*_*t*_) < 0). Perseveration for all other actions, *c*_*t*__+1_(*a* ≠ *a*_*t*_), is 0 (Fig. 2a, right). The outputs of both modules, ‘reward logits’ *Q*_*t*__+1_ and ‘action logits’ *c*_*t*__+1_, are combined additively before sampling the action *a*_*t*__+1_ that is taken on the next trial (for the model details and equations, see ‘Model architectures’ in Methods).

Best RL is a prime example of a classic handcrafted cognitive model: each mechanism is clearly defined by simple equations, which are modified by just a small number of interpretable model parameters (for example, the inverse decision temperature, *β*). However, these constraints limit the model’s expressivity and potentially its ability to capture human behaviour. To assess whether this is the case, we compared Best RL to a highly expressive ‘Vanilla RNN’, which can employ a large number of free parameters to model increasingly complex functions. At the core of Vanilla RNN is a recurrent memory module that allows the model to directly share its high-dimensional hidden-layer activations, the latent state **s**_*t*_, with itself on subsequent trials (for details and equations, see ‘Model architectures’ in Methods; Fig. 2c). This allows Vanilla RNN to rely on a rich and flexible memory of past trials when making choices. Compared with Best RL, Vanilla RNN has the additional advantage of processing all inputs (*a*_*t*_, *r*_*t*_ and **s***s*_*t*_) jointly, allowing it to identify arbitrarily complex interactions between them. (Besides the basic RNN architecture, we also fitted more sophisticated sequence models such as long short-term memory networks (LSTMs^45^ and transformers^46^, which led to qualitatively similar results; see ‘Additional model fits’ in the Supplementary Information.)

We fit both models to our human data using a cross-entropy loss (equivalent to negative log-likelihood) that quantified how well each model predicted human choices. Note that the models were not trained to find the reward-maximizing policy for the task but to recreate the observed human data as accurately as possible. This approach is sometimes referred to as ‘system identification’ in engineering^47^ or ‘behavioural cloning’ in machine learning^48,49^. We evaluated all models by cross-validating over participants. This amounts to using a subset of participants to identify the algorithm that best predicted the behaviour of the remaining participants, who completed a different set of task schedules. We trained all models on the same 80% of participants (690 participants; 3,302 task blocks) and tested all models’ predictive performance on the same held-out 10% (86 participants; 413 blocks), using the remaining 10% (86 participants; 419 blocks) to select the best hyperparameters for each model (for example, the number of hidden units). Training, validating and testing on different sets of participants eliminates the risk that increasingly flexible models overfit to the training data, and it makes models with different numbers of free parameters directly comparable (see ‘Model training’ in Methods and Supplementary Table 1). We confirmed that different models were behaviourally distinguishable by generating synthetic behaviour from each model and confirming that the correct model could be identified; this was generally possible because less-flexible models were unable to imitate more-flexible ones (Supplementary Fig. 1). In terms of model comparison, we found that Vanilla RNN predicted the behaviour of unseen participants substantially better than Best RL, correctly anticipating 68.3% (95% CI, (66.9%, 69.7%)) of unseen participants’ choices, compared with just 60.6% (95% CI, (59.2%, 62.0%)) by Best RL (chance is 25%; Vanilla RNN versus Best RL, paired *t*-test: *t*_412_ = 28.9, *P* < 0.001, *d* = 1.39; Fig. 2d). This confirms that, as expected, Vanilla RNN can predict human behaviour more accurately than the best classic RL model. A data sensitivity analysis (Supplementary Fig. 7a) showed that Vanilla RNN’s advantage became increasingly prominent for increasing sizes of training data, indicating that collecting more data can improve the extraction of systematic behavioural variance.

Next, we created a series of models that interpolate between the extremes of Best RL and Vanilla RNN. We first created a hybrid model that inherits the architecture of Best RL (Fig. 2a) but replaces its handcrafted equations with flexible ANNs (Fig. 2b). As in Best RL, the reward module is responsible for updating the chosen action’s *Q*-value at each time step. The module has access to the previous reward *r*_*t*_ (for example, ‘received 70 points’) and value *Q*_*t*_(*a*_*t*_) (for example, ‘expected 50 points’), but not the identity of the chosen action *a*_*t*_ (for example, ‘pressed the D key’). In turn, the action module updates the chosen action’s perseveration indicator, for which it has access only to the previous action *a*_*t*_ (for example, ‘pressed the D key’). Unlike Best RL, both modules use flexible ANNs to map their respective inputs to the corresponding updated output. This model, which we call ‘RL-ANN’, is motivated by the insight that Best RL’s strictly linear *Q*-value updates (Fig. 2e) (in conjunction with Best RL’s restrictive perseveration mechanism; Supplementary Fig. 6) might be insufficient to capture human learning. For example, existing models propose that value updates might depend on reward in various nonlinear ways^19,50^, but the strictly linear *Q*-learning model does not account for possibilities like these. Similarly, values might depend nonlinearly—or even non-monotonically—on previous values and rewards, but the model does not express this possibility. By replacing Best RL’s linear update equations with generic ANNs, we were able to simultaneously test all nonlinear model variants of this kind, without the necessity of specifying each one by hand. During training, RL-ANN’s value and action modules have the flexibility to acquire update rules of any functional form and will settle on the one that allows the model as a whole to best match human behaviour. In this sense, RL-ANN represents a whole class of cognitive models: any model that shares Best RL’s architecture can in principle be instantiated by RL-ANN, independent of the specific functional form of its updates (for an example, see Supplementary Fig. 1). When we assessed how well RL-ANN predicts the behaviour of unseen participants, however, this added flexibility did not close the gap to Vanilla RNN (RL-ANN: 60.8%; 95% CI, (59.4%, 62.3%); Vanilla RNN: 68.3%; 95% CI, (66.9%, 69.7%); paired *t*-test: *t*_412_ = 32.7, *P* < 0.001, *d* = 1.35; Fig. 2d; also see Supplementary Fig. 3 for additional variants of Best RL). This suggests that there is no RL-like model—defined as a model that shares Best RL’s cognitive architecture, albeit with complete flexibility in terms of the implemented functions—that can predict human behaviour on our task as well as Vanilla RNN. This shows that there exist no modifications to Best RL’s update rules that improve the prediction of human task behaviour.

Perhaps surprisingly, RL-ANN did not significantly improve predictions compared to Best RL (RL-ANN: 60.8%; 95% CI, (59.4%, 62.3%); Best RL: 60.6%; 95% CI, (59.2%, 62.0%)); paired *t*-test: *t*_412_ = 1.54, *P* = 0.12, *d* = 0.70), suggesting that Best RL’s original update rules might already be the best in its class. To see if this was the case, we conducted two analyses. We first inspected RL-ANN’s learned update functions and compared them to their handcrafted counterparts in Best RL. This analysis can reveal whether among all possible mechanisms RL-ANN could implement, human behaviour lent the most support to the special case of Best RL. We visualized Best RL’s *Q*-value update (Fig. 2e) by calculating the updated values *Q*_*t*__+1_(*a*_*t*_) for every combination of inputs (0 < *r*_*t*_ < 100 points; 0 < *Q*_*t*_(*a*_*t*_) < 1), using the standard *Q*-value equations (Fig. 2a, left; see ‘Model analysis’ in Methods). We also visualized RL-ANN’s *Q*-value update by extracting the fitted reward module and probing it across its range of inputs (0 < *r*_*t*_ < 100 points; *Q*_*t*_(*a*_*t*_) between the 5th and the 95th percentile of observed *Q*-values), while recording its outputs *Q*_*t*__+1_(*a*_*t*_). Indeed, RL-ANN showed an update rule that was monotonic and approximately linear in both *r*_*t*_ and *Q*_*t*_, similar to Best RL (Fig. 2f), suggesting that human behaviour was best approximated by an algorithm very similar to RL. The corresponding analysis of the action module is shown in the Supplementary Results (Supplementary Fig. 6a). Second, we generated and analysed synthetic behavioural data from both Best RL and RL-ANN, assessing whether the slight differences in the update rule between both would lead to meaningful differences in behaviour. We used each trained model to simulate a behavioural dataset with the same characteristics as the human dataset (the same sample size, reward schedules and train–test–validation split; see ‘Model analysis’ in Methods). We found that behavioural datasets from both models were qualitatively similar (Supplementary Figs. 10 and 11) but differed from human behaviour (Fig. 4). Thus, even when given the opportunity to learn new, more expressive operations for updating *Q*-values, RL-ANN approximately recovers the simple solution found in classic RL models and, like them, falls short in predicting human behaviour (Fig. 2d).

**Fig. 3:**
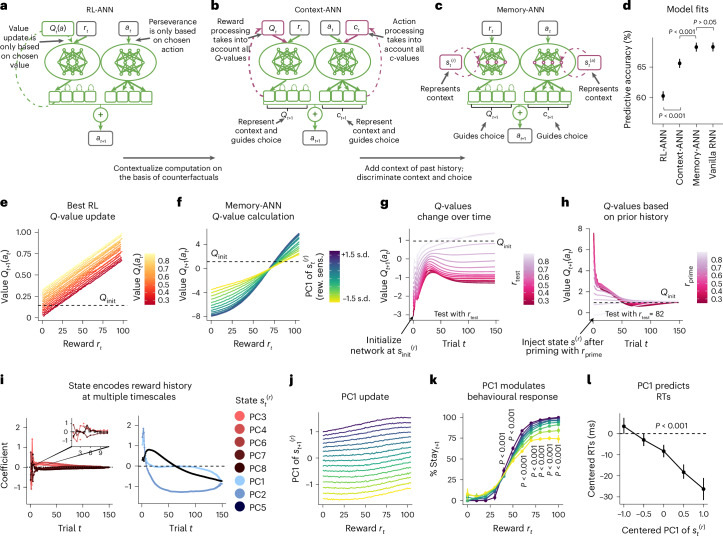
Interpretability. **a**–**c**, Model architectures. Context-ANN (**b**) and Memory-ANN (**c**) were incremental extensions of RL-ANN (**a**). **d**, Model fits. Both Context-ANN and Memory-ANN provided significant improvements, with Memory-ANN not differing from the predictive accuracy of Vanilla RNN, according to two-sided *t*-tests (see the main text for statistics). The data are presented as mean values over held-out blocks (*n* = 413) plus/minus s.e.m. **e**–**i**, Model algorithms. Panel **e** shows the Best RL *Q*-value update (details in Fig. 2e). Panel **f** shows the Memory-ANN *Q*-value calculation. Memory-ANN implements a monotonic but nonlinear, sigmoidal function (Supplementary Fig. 8b), mapping higher rewards onto higher values. The colours show the first principal component (PC1) of the memory state $\bf{s}_{t}^{(r)}$, which we interpret as reward sensitivity. The reference point (*Q*_init_) indicates the initial *Q*-value, towards which values of non-chosen actions gradually decayed. As shown in **g**, *Q*-values change over time. When iteratively probed with the same reward magnitude *r*_test_ (colour), Memory-ANN produces different *Q*-values *Q*_*t*__+1_(*a*_*t*_) depending on the trial *t*. Panel **h** shows how *Q*-values are based on prior history. Different states **s**^(*r*)^ were obtained by subjecting Memory-ANN to 150 different iterations of observing reward *r*_prime_ (colour). Injecting different states **s**^(*r*)^ led to wide variations in the response to the same *r*_test_, which was given for 150 time steps. As shown in **i**, the state encodes the reward history at multiple timescales. Coefficients were obtained from regressing past rewards *r*_1:*t*−1_ against each PC of $\bf{s}_{t}^{(r)}$ (see ‘Behavioural analyses’ in Methods). Some PCs showed sensitivity to the most recent history of rewards, while others showed sensitivity to the long-term history of rewards. **j**, PC1 update. The memory representation $\bf{s}_{t}^{(r)}$ is affected by incoming rewards *r*_*t*_. PC1 of $\bf{s}_{t}^{(r)}$ exhibits monotonic, near-linear, incremental integration. **k**,**l**, Behavioural relevance. PC1 modulates the behavioural response (**k**). The mapping from reward magnitudes to action reselection (‘Percent stay’) is modulated by PC1 of $\bf{s}_{t}^{(r)}$ (colour). *P* < 0.001 in paired, within-participant, Bonferroni-corrected, two-sided *t*-tests comparing stay frequency between PC1 ≤ 0 and PC1 > 0, separately for each bin of reward magnitudes (*x* axis). PC1 also predicts response times (**l**). The relation between PC1 of $\bf{s}_{t}^{(r)}$ (fitted to human behaviour) and response times (RTs) (log-transformed; both mean-centred within blocks) is shown. The data are presented as mean values over all blocks (*n* = 4,134) for each PC1 bin, plus/minus 95% bootstrapped CIs. *P* < 0.001 in mixed-effects linear regression (see the main text).

Our second hybrid model aims to address this issue by generalizing the architecture further and considering a broader space of models. It is inspired by the finding that learning is affected not only by properties of the chosen option but also by those of options that were available but not chosen, a notion commonly referred to as ‘context’^18,19,21,51,52^. For example, an action that won 50 points might be processed differently depending on whether other available actions were expected to win 10 points or 90. To allow for this possibility, we provided the ‘Context-ANN’ model with additional connections that allow learned information about unchosen actions to modify the learning rule (Fig. 3b). Context-ANN’s reward module receives as additional input its own value estimates *Q*_*t*_ (the previous trial’s *Q*-values of all four actions); the action module receives as additional modulatory input *c*_*t*_ (the previous trial’s perseveration indicators for all four actions). These modulatory inputs allow Context-ANN to adopt any learning algorithm that can be expressed as a function of the primary input (*r*_*t*_, *a*_*t*_) and the corresponding choice variables for all available actions (*Q*_*t*_, *c*_*t*_). In model comparison, Context-ANN fit human behaviour substantially better than RL-ANN, increasing the percentage of correctly predicted choices from 60.8% (95% CI, (59.4%, 62.3%)) to 65.4% (95% CI, (63.9%, 66.9%); paired *t*-test: *t*_412_ = 28.3, *P* < 0.001, *d* = 1.27; Fig. 3d). Each module played a unique role in improving the prediction accuracy (Supplementary Tables 3 and 4). Nevertheless, Context-ANN still fell short of Vanilla RNN (68.3%; 95% CI, (66.9%, 69.7%); paired *t*-test: *t*_412_ = 16.8, *P* < 0.001, *d* = 0.83), indicating that the inclusion of context processing was not sufficient to capture human behaviour on our task and that an even more flexible architecture is required.

We hence turned to the role of memory processing, testing whether a model that can retain a richer representation of the past can explain human behaviour better than previous models. Indeed, several studies have shown that both recent^53^ and distant^13,17^ outcomes affect human learning in ways that cannot be explained by incremental updating alone. It has also been suggested that humans keep track of additional latent variables beyond *Q*-values—for example, remembering past prediction errors to adapt the future speed of learning^54^. (We implemented several versions of such variable-learning-rate models, which showed slightly better performance than Best RL but still fell far short of Vanilla RNN; see ‘Model architectures’ in Methods and Supplementary Results). To test whether the ability to retain richer representations of the past is crucial to explain learning in our task, we created our final hybrid model: Memory-ANN. Whereas Context-ANN receives the modulatory inputs *Q*_*t*_ and *c*_*t*_ to account for unchosen actions, Memory-ANN requires inputs that have potential access to the entire task history and could represent any summary statistic thereof, including high-dimensional and nonlinear ones. The latent states of an RNN have precisely these properties. We hence replaced the reward module’s inputs, *Q*_*t*_ and *Q*_*t*_(*a*_*t*_), with the activities of the reward module’s hidden units from the previous time step, which we denote $\bf{s}_{t}^{(r)}$ (this turns the reward ANN into a reward RNN). Likewise, we replaced the action module’s input *c*_*t*_ with the previous activities of its hidden units, $\bf{s}_{t}^{(a)}$ (turning the action ANN into an action RNN; Fig. 3c). These modifications have the effect of explicitly separating memory variables ($\bf{s}_{t}^{(r)}$ and $\bf{s}_{t}^{(a)}$) from choice variables (*Q*_*t*_ and *c*_*t*_), which in previous models were assumed to be identical. Hence, Memory-ANN has the ability to express a wide range of memory-based learning models that are based on modulating reward (and action) processing on the basis of any learned features of the reward (and action) history. Note, however, that Memory-ANN is still more constrained than Vanilla RNN: the same update applies regardless of which action is being updated, the values of all unchosen actions decay strictly exponentially, reward processing does not have access to past or present actions and vice versa for action processing, and the outputs of reward and action processing are combined by simple addition. Memory-ANN improved the prediction of human behaviour substantially compared with Context-ANN (Context-ANN: 65.4%; 95% CI, (63.9%, 66.9%); Memory-ANN: 68.3%; 95% CI, (66.9%, 69.7%); paired *t*-test: *t*_412_ = 17.9, *P* < 0.001, *d* = 0.95; Fig. 3d). Most importantly, Memory-ANN’s predictions were not significantly different from those of Vanilla RNN, the most general model we tested (Memory-ANN: 68.3%; 95% CI, (66.9%, 69.7%); Vanilla RNN: 68.3%; 95% CI, (66.9%, 69.7%); paired *t*-test: *t*_412_ = 0.32, *P* = 0.75, *d* = 0.14). This indicates that Memory-ANN extracted all systematic variance in the dataset that can be extracted by an RNN, suggesting that its architectural constraints (Fig. 3c) identified relevant biases in human behaviour. Indeed, there was no constraint whose removal improved model prediction (Supplementary Tables 4, 5, 7 and 8). Taken together, these results suggest that our participants performed the task by creating rich memories of reward and action history and used them to guide reward learning.

What mechanisms underlie the learning processes in Memory-ANN? To answer this question, we inspected the functions learned by the neural network modules during model fitting. We first considered reward processing, evaluating the reward module by probing it across its range of inputs (*r*_*t*_ and $\bf{s}_{t}^{(r)}$) while recording its outputs *Q*_*t*__+1_(*a*_*t*_) (see ‘Model analysis’ in Methods). We found that the reward module maps rewards *r*_*r*_ onto new values *Q*_*t*__+1_(*a*_*t*_) in a monotonic, roughly sigmoidal way (Fig. 3f and Supplementary Fig. 8b). Notably, the reward module does not have access to previous values *Q*_*t*_(*a*_*t*_) (nor can it reconstruct them using its hidden state input $\bf{s}_{t}^{(r)}$), which means that Memory-ANN does not take into account previous values *Q*_*t*_(*a*_*t*_) when calculating new values *Q*_*t*__+1_(*a*_*t*_). This is in stark contrast to most RL models, which posit that values are learned incrementally. Instead, Memory-ANN simply maps large rewards onto large *Q*-values and small rewards onto small *Q*-values, without calculating reward prediction errors or incremental updates. If Memory-ANN’s latent state $\bf{s}_{t}^{(r)}$ was fixed over time (Supplementary Fig. 12a), this simple mapping mechanism would lead to somewhat rigid choice behaviour (Supplementary Fig. 12b). However, the flexibility of the latent state enables adaptive choices: $\bf{s}_{t}^{(r)}$ follows a stereotypical trajectory over the time course of a task (Supplementary Fig. 5f), which leads to a gradual change in the assignment of *Q*-values to rewards as the task progresses. To assess this, we initialized a fresh reward module and probed it with sequences of identical rewards, recording the resulting *Q*-values. Across the range of rewards, earlier trials lead to smaller *Q*-values than later ones, which can support a behavioural shift from more ‘exploratory’ to more ‘exploitative’ choices (Fig. 3g and Supplementary Results). $\bf{s}_{t}^{(r)}$ also adapts the calculation of future *Q*-values by encoding complex moments summarizing the history of rewards. We forced a fresh reward module into several extreme states by priming it with different reward sequences and tested its responses to a new reward. This reward elicited tremendously different *Q*-values depending on the injected state, an effect that took up to several dozen trials to disappear (Fig. 3h). We finally causally probed the role of state **s**^(*r*)^ by injecting activity into different principal components (PCs), observing the corresponding short- and long-term perturbations in the calculation of *Q*-values (Supplementary Fig. 12e), and testing the effects of individual trigger rewards (Supplementary Fig. 12c) or reward sequences (Supplementary Fig. 12d) on state **s**^(*r*)^.

We next identified the mechanisms by which $\bf{s}_{t}^{(r)}$ biases the calculation of *Q*-values. We conditioned the trained Memory-ANN on each participant’s action sequence to obtain, for each participant, the trial-by-trial sequence of latent variables *Q*, *c*, **s**^(*r*)^ and **s**^(*a*)^ (Supplementary Fig. 5a–g), and applied principal component analysis to **s**^(*r*)^. The first component (PC1) modified the gain of the sigmoidal mapping from rewards to *Q*-values, effectively controlling the sensitivity of *Q*-values to reward magnitude (Fig. 3f). At high gains (blue), even small reward differences lead to large differences in *Q*-values, whereas at small gains (yellow), large reward differences are required to produce moderate differences (Fig. 3f). We therefore interpreted PC1 as tracking the model’s current sensitivity to reward. Confirming this interpretation, we found that large values of PC1 are associated with a high probability of repeating a choice that led to a large reward and a small probability of repeating a choice that led to a small reward, while low values of PC1 are associated with a shallower relationship (Fig. 3k). The value of PC1 also correlated with participants’ response times, showing that a model-derived variable predicted behaviour in a dimension that was not included in the training data (mixed-effects regression, slope = −0.968.9, *z* = −13.3, *P* < 0.001; Fig. 3l). These modulations occurred within participants, showing that fluctuations in reward sensitivity captured gradual changes in participants’ behavioural strategies, rather than individual differences between participants. (For more discussion of individual differences, see Supplementary Results.) Subsequent PCs affected the gain, range, bias and scale of the sigmoid (Supplementary Fig. 8). This mechanism enables Memory-ANN to flexibly adapt its behaviour to the current reward context. The corresponding analysis for Memory-ANN’s action module is shown in the Supplementary Results (Supplementary Fig. 6a).

How does $\bf{s}_{t}^{(r)}$ represent the past history? We first determined how new information alters existing representations. We probed the reward module across its range of inputs (*r*_*t*_ and $\bf{s}_{t}^{(r)}$), this time collecting the latent state $\bf{s}_{t+1}^{(r)}$ as the output. We found that PC1 (reward sensitivity) integrated rewards in a monotonic, near-linear way, increasing slightly after big rewards and decreasing slightly after small ones (Fig. 3j). Several other state PCs showed similar monotonic, near-linear integration patterns, exhibiting steeper (for example, PC3 and PC4) or shallower slopes (for example, PC5) (Supplementary Fig. 8). This supports the notion that $\bf{s}_{t}^{(r)}$ integrates new rewards using parallel update rules with a variety of integration timescales (see also Supplementary Fig. 12). We next assessed the contents of the representations, using a decoding analysis. For each delay *i*, we used lagged regression to predict each state PC from the reward *r*_*t*−*i*_. We found that a subset of PCs showed large regression weights to just a handful of the most recent rewards, consistent with the idea that these PCs track individual recent outcomes while being insensitive to all earlier events (Fig. 3i, left). Some other PCs were sensitive to the entire history of rewards, potentially providing a baseline for how reward-rich the environment is overall, and whether this is changing for better or for worse (Fig. 3i, right). These results were consistent across multiple independent runs of Memory-ANN (Supplementary Fig. 2) and were recoverable when Memory-ANN was fit to synthetic data (Supplementary Fig. 1). These findings indicate that Memory-ANN flexibly modulates the mapping from rewards and *Q*-values, continuously adjusting to the time on task and the reward history.

**Fig. 4:**
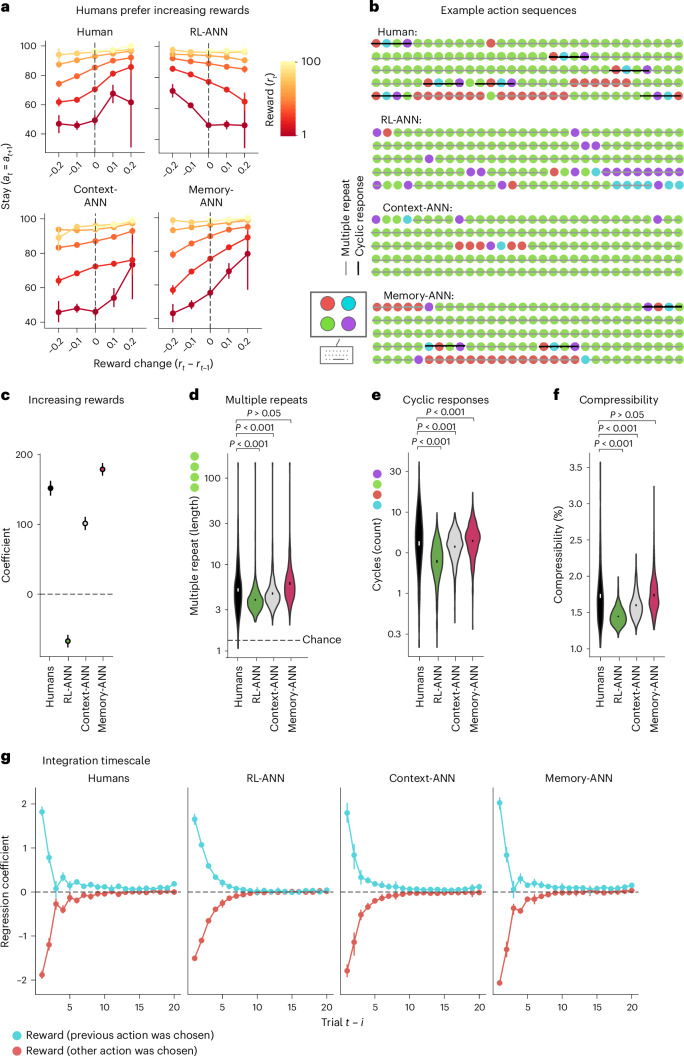
Behavioural model validation. **a**–**g**, Comparison of human and simulated model behaviour. As shown in **a**, humans prefer increasing rewards. Humans (top left) repeated a choice (‘stayed’; *y* axis) more often when the rewards for this choice had increased (positive reward change; *x* axis) rather than decreased (negative reward change) on the previous two trials. Best-RL (Supplementary Fig. 11a) and RL-ANN (top right) showed the inverse pattern, whereas Context-ANN and Memory-ANN qualitatively reproduced the effect. The data are presented as mean values over blocks (*n* = 4,134) for each reward change bin, plus/minus 95% bootstrapped CIs. Panel **b** shows example action sequences. These are raw sequences of chosen actions (coloured circles) from humans and models performing the reward schedule shown in the top row of Supplementary Fig. 5a. Humans showed two common patterns: multiple repeats, extended periods of the same action (grey lines); and cyclic responses, sets of four sequential trials in which each action was sampled once (black lines). Panel **c** shows the effect of reward change on stay probability (see **a**). The data are presented as the regression coefficients from the model stay ~ reward × reward_change (*n* = 862 participants), plus/minus standard errors of the coefficient estimates. As indicated in **d**, humans showed longer sequences of identical actions (average length, 6.9; 95% CI, (6.1, 7.6)) than expected by chance (chance length, 1.3; *t*_861_ = 14.6, *P* < 0.001, *d* = 0.50) or seen in RL-ANN (average length, 4.5; 95% CI, (3.9, 5.1); *t*_861_ = 9.4, *P* < 0.001, *d* = 0.32) and Context-ANN (average length, 5.5; 95% CI, (4.8, 6.1); *t*_861_ = 5.4, *P* < 0.001, *d* = 0.19). Memory-ANN sequence length did not differ from that of humans (average length, 7.5; 95% CI, (6.8, 8.3); *t*_861_ = −1.8, *P* = 0.075, *d* = 0.06). As shown in **e**, human choices contained more cyclic sequences than synthetic data (human mean, 5.37; 95% CI, (5.01, 5.68); RL-ANN mean, 2.68; 95% CI, (2.59, 2.76), *t*_861_ = 10.9, *P* < 0.001, *d* = 0.37; Context-ANN mean, 3.87; 95% CI, (3.77, 3.97), *t*_861_ = 7.77, *P* < 0.001, *d* = 0.26). Memory-ANN produced the qualitatively closest number of cyclic sequences compared to humans (Memory-ANN mean, 4.62; 95% CI, (4.48, 4.76), *t*_861_ = 5.7, *P* < 0.001, *d* = 0.19). As shown in **f**, we used the Lempel–Ziv–Welch algorithm to compress human and model action sequences (see ‘Behavioural analyses’ in Methods), quantifying systematic temporal structure. Human sequences were substantially more compressible than those of RL-ANN and Context-ANN (human mean, 1.73; 95% CI, (1.70, 1.76); RL-ANN mean, 1.45; 95% CI, (1.44, 1.45), *t*_861_ = 20.48, *P* < 0.001, *d* = 0.70; Context-ANN mean, 1.60; 95% CI, (1.59, 1.61), *t*_861_ = 9.47, *P* < 0.001, *d* = 0.322). Memory-ANN compressibility did not differ from that of humans (mean, 1.74; 95% CI, (1.72, 1.76), *t*_861_ = 0.69, *P* = 0.49, *d* = 0.02). In **d**–**f**, the data are presented as violin plots of the raw data distribution (*n* = 862 participants) and error bars indicating 95% CIs of the mean. *P* < 0.001 in paired, two-sided *t*-tests. Panel **g** shows the integration timescale. Weights of trial-history regression models trained to predict future choices on the basis of past choices and rewards (see ‘Behavioural analyses’ in Methods) are plotted. Only Memory-ANN reproduced the patterns seen in human behaviour qualitatively. The data are presented as mean values over participants (*n* = 862) for each trial, plus/minus 95% bootstrapped CIs. (The corresponding plots for Simple RL, Best RL and Vanilla RNN for all these measures are shown in Supplementary Figs. 10 and 11).

Finally, we tested whether Memory-ANN captured qualitative features of human behaviour that more restricted models were unable to capture^55^. We used each fitted model to simulate task behaviour ‘open-loop’ (without knowledge of human choices), and on the same tasks as humans (see ‘Model analysis’ in Methods). First, we sought a behavioural signature of the history-dependent processing of reward sequences. For this, we considered pairs of trials in which the same action was selected twice in a row, and we quantified the tendency to select that action again on the following trial as a function of the change in reward magnitudes (Fig. 4a). Best-RL (Supplementary Fig. 11a) and RL-ANN preferred actions for which the second-most-recent reward *r*_*t*−*1*_ was larger than the most recent reward *r*_*t*_ (colour), on which we conditioned. This arises because these models make choices on the basis of running averages, and a larger reward in the past increases this average. Humans, in contrast, preferred actions whose second-most-recent rewards were lower^56,57^, as if anticipating that a recent increase in reward magnitudes will continue in the future. Only Context-ANN and Memory-ANN reproduced this effect qualitatively (Fig. 4a,c). This shows that memory representations need to contain information about unchosen actions or task history to capture how participants modify their responses. Second, we assessed behavioural patterns related to the history-dependent processing of actions. We focused on stereotyped action sequences, such as multiple repeats (AAAA) and cyclic responses (ABCD), in which the time horizon extends for multiple trials^58^. Memory-ANN was able to capture the strong human preference for such multi-trial patterns, while no other model was able to do so (Fig. 4b,d–f). The prevalence of these behavioural motifs implies that human participants committed to stretches of exploiting an action they believed was best (AAAA), interspersed with brief episodes of systematically exploring whether a different action might be better (ABCD)^59^. We then characterized the overall structure within the observed choice patterns, computing the compressibility of all action sequences using a standard algorithm (see ‘Behavioural analyses’ in Methods) and comparing humans to model predictions (Fig. 4f). Only Memory-ANN achieved a similarly high compression ratio as humans; Context-ANN showed intermediate compressibility, and RL-ANN showed the lowest compressibility. This reveals that the choices of humans and of Memory-ANN had structured relationships with other choices nearby, which was less the case in simpler models. Finally, we assessed the history dependence of actions using lagged regression^60^. We found that participants showed shallow and non-monotonic history dependence that was reproduced by Memory-ANN but not by other models (Fig. 4g). Memory-ANN hence captures a range of patterns that are characteristic of human behaviour, including many that violate classic models. While some of these patterns have been described in the past^14,56–59,61^, they have not previously been captured in a single model. It is a challenge in computational cognitive science that the identification of new patterns often leads to the creation of idiosyncratic model features and a multiplication of model architectures, rather than consolidation in a single framework.

## Discussion

In psychology and neuroscience, reward-learning behaviour is commonly understood using computational models based on *Q*-learning, in which memory consists entirely of a set of incrementally updated decision variables. We have shown that this family of models cannot adequately account for reward-guided learning in humans, using a large dataset from a classic reward-learning task and a systematic model comparison approach that integrates deep neural networks into classic cognitive architectures. We identify instead a model that contains both decision variables that drive choice directly and a set of latent memory variables that modulate the update of these decision variables but do not directly drive choice. These memory variables track a complex history of rewards and choices over multiple timescales. We show that this model captures human behaviour in detail, both reproducing a number of intricate features of the dataset and matching generic neural networks in quantitative quality of fit. At the same time, it is interpretable as an algorithmic model of human reward learning.

Recent work implicitly recognizes the complexity of how humans use memory in reward-learning tasks, highlighting that learning processes often operate at multiple different timescales. This has been framed as a multiplicity of memory mechanisms^13,14,53,62–65^ and is consistent with evidence that the brain represents task-history information at a diversity of timescales^26,66–68^. Memory beyond decision variables is also present in several handcrafted models of human reward learning. For example, Bayesian inference models^37,69,70^ track a measure of the model’s uncertainty that creates non-Markovian dependencies between choice variables, variable-learning-rate models^71,72^ track a measure of environmental volatility, and actor-critic models^73,74^ and reference-point models^75^ track an action-independent measure of expected value. However, all these models are based on handcrafted equations, and the ones we have tested here fall short compared with more flexible ANN-based models. Memory-ANN reveals that learning at different timescales is supported by a flexible recurrent memory system that is one step removed from behavioural choice, and it shows that the way in which observed outcomes are mapped to future choices is a complex, yet interpretable, function of task history.

The cognitive architecture of Memory-ANN is modular in two ways. First, reward-based learning and action-based learning are divided into two parallel modules. This idea has origins in early work on the psychology of learning—for example, in the distinction between Thorndike’s^76^ law of effect (actions that lead to good outcomes should be repeated) and law of exercise (actions that have been taken in the past should be repeated). A separation of reward-based from action-based learning is present in a number of computational models of behaviour^5,43,70,77^, and evidence from neuroscience suggests that the brain may incorporate such modularity^78–81^. These models typically imagine that action-based learning takes the form of perseveration, in which actions that have been taken in the past are more likely to be taken in the future^43^, and that reward-based learning takes the form of incremental RL^1,2^. Memory-ANN retains the basic separation between reward-based and action-based learning but allows for each module to implement substantially more sophisticated mechanisms. This uses Memory-ANN’s second kind of modularity: both reward-based and action-based learning are divided into a ‘deep’ memory component, which learns rich hidden representations of the past but does not drive choice, and a ‘shallow’ choice component that guides action selection. This architecture shares features with models of more complex reward-learning tasks, many of which draw on hierarchical cognitive architectures^6,82–85^. Evidence from neuroscience also supports the idea of a gradient of abstraction in the neural architecture^82,86,87^. Our results suggest that humans may use hierarchically structured algorithms even in superficially simple reward-learning tasks.

One limitation of the current work is a lack of focus on individual differences. We fit a single model to the whole population, which allows us to infer the likely mechanisms that characterize the behaviour of all participants but does not provide insight into individual differences between them. Others have modelled individual differences within RNN-based frameworks^31,88^, and similar approaches could be used to extend the current work. However, RNN-like models implicitly capture individual differences even when they are not modelled explicitly^89^, which means that in principle, some of our results concerning differential performance between Memory-ANN and Best RL might reflect the network better capturing aspects of between-participant differences, rather than (as we interpret it) improved modelling of the progression of learning within each participant. While additional analyses ruled out the possibility that this difference between the models accounts for our key results (for example, that Memory-ANN outperforms Best RL and that aspects of its architecture and latent state dynamics capture within-participant learning), it remains possible that some of our conclusions reflect a contribution of both between- and within-participant effects. Additional work, both experimental and analytical, will be required to fully tease apart these possibilities. Overall, this direction offers intriguing new prospects for studying individual differences as well as the dynamic fluctuations that occur within individuals over time (Supplementary Information).

Science faces a theory discovery problem: it is fundamentally more difficult to create new models than to evaluate existing ones^90,91^. In psychology and neuroscience, new laboratory technologies have enabled scientists to collect larger datasets than ever before, a development that might provide new solutions to this problem^5,32,92–95^. We used a combination of hypothesis-driven architecture search and data-driven function approximation^36^ to successfully identify a predictive yet interpretable model of human reward-based learning. With the rich tradition of classic cognitive modelling providing the theoretical framework to guide our model search, machine learning tools contributed the ability to approximate any functional form on the basis of sufficient data. This approach allowed us to compare the most relevant model classes in the most general case. The same approach could be applied to a wide range of open questions, both within the cognitive sciences and beyond. There is a ubiquitous need for models that can capture the complexity in rich datasets and also provide interpretable explanations.

## Methods

### Dataset

#### Participants

We recruited 880 participants on Prolific (app.prolific.co). No statistical methods were used to predetermine the sample size, but our sample size is orders of magnitude larger than those of most traditional lab-based human experimental studies and similar to those reported in previous publications focused on large-scale experiments^36,39,96,97^. In agreement with the ethical guidelines of the Google DeepMind Human Behavioral Research Ethics Committee, all participants were local to the UK and fluent in English. The participants provided informed consent and were paid at a rate of 12 pounds per hour; there was no performance-based bonus payment. The study was not preregistered.

#### Experimental procedure

The participants completed one training block and several testing blocks of our bandit paradigm (see below), each using different visual stimuli. After each block, the participants were truthfully informed how many points they had won, how many points they could have won (the sum of points from each trial’s best choice option) and how many points they would have won by choosing randomly (the average points of all choice options). At the end of the study, the participants were asked for their highest level of education and offered the opportunity to voice thoughts and concerns. The experimental task was written using jsPsych^98^ and served on cognition.run.

#### Exclusion criteria

Eighty participants were asked to complete one training and three testing blocks of 150 trials each. The remaining 800 participants were asked to complete one training block of 50 trials and five testing blocks of 150 trials, for a total of 4,240 task blocks. Four participants in the first (5%) and 14 participants (1.75%) in the second sample failed to finish the experiment and were excluded, leading to an initial sample of 880 − 18 = 862 participants who collectively finished (80 − 4) × 3 + (800 − 14) × 5 = 4,158 task blocks. We further excluded blocks in which participants missed more than 15 of the 150 trials (10%), 24 blocks in total (0.58%). Hence, our final dataset comprised 4,134 blocks (with 617,871 valid trials) from 862 participants. Of these 862 participants, 858 (99.5%) provided valid demographic information: 341 (39.7%) were female, and 517 (60.3%) were male; the average age was 39.7 years, with a range of 18–88 and a standard deviation of 13.1 years.

### Task

The participants performed a classic four-armed drifting bandit task^37,99^. On each trial *t* of this task, participants chose one of four bandits and observed the corresponding reward *r*_*t*_. At the first trial *t* = *1*, each arm was initialized independently and uniformly at random between 1 and 100 points. The mean reward *μ*_*t*,*i*_ at each trial *t* and arm *i* was determined by a Gaussian random walk that evolved according to standard deviation *σ*_d_ and centrality *λ*:

$${\mu }_{t,i}\sim {\rm{N}}(\lambda \times {\mu }_{t-1,i}+(1-\lambda )\times 50,{\sigma }_{{\rm{d}}})$$

The actual reward *r*_*t*,*i*_ observed by participants was sampled from a Gaussian distribution with mean *μ*_*t*,*i*_ and standard deviation *σ*_o_:

$${r}_{t,i}\sim {\rm{N}}(\,{\mu }_{t,i},{\sigma }_{{\rm{o}}})$$

Following prior work^37,99^, we used *λ* = 0.9836, *σ*_d_ = 2.8 and *σ*_o_ = 4. Unlike prior work^37,99^, we created a new reward schedule for each participant for each task to increase the behavioural variation in the dataset and facilitate the fitting of neural network models.

On each trial, the participants saw four visual stimuli on the screen, one representing each bandit (Fig. 1d). Each bandit was presented in the same location on each trial, but new stimuli were used on each task iteration, and their positions were randomly shuffled between participants. Participants had four seconds to select a bandit using the keys ‘D’, ‘F’, ‘J’ and ‘K’. When participants failed to make a response within this time window, they were encouraged to respond faster on the next trial and reminded of the response keys. The participants were also told that they had received zero points for that trial. Only a very small percentage of trials in the final sample were missed (0.36%). When participants made a valid selection, the chosen bandit remained on the screen for 400 milliseconds while the others disappeared. The trial outcome was then presented in addition to the chosen bandit (for example, ‘You won 79 points.’). After another 800 milliseconds, an inter-trial interval of 500 milliseconds began, after which the next trial started.

### Behavioural analyses

#### Task performance

We first aimed to assess participant performance. The raw number of points is not a good measure of performance because each task block is based on a different reward schedule (see above), and hence the same number of points can indicate good or bad performance. To obtain a performance measure that is comparable between blocks, we calculated relative rewards. The relative reward *r*_rel,*t*_ is the number of points *r*_*t*_ obtained on trial *t*, normalized between the maximum number of points available on that trial (max(*p*_*t*_)) and the number of points expected on that trial by random selection (mean(*p*_*t*_)):

$${r}_{\mathrm{rel},t}=\frac{{r}_{t}-\mathrm{mean}(\,{p}_{t})}{{\mathrm{max}}({p}_{t})-\mathrm{mean}(\,{p}_{t})}$$

Averaging *r*_rel,*t*_ across all trials *t* gives the relative reward of a block *r*_rel_, shown in Fig. 1f. A block’s relative reward would be 1 if a participant chose the best bandit on each trial (which is impossible); the relative reward is close to 0 when a participant chooses randomly and smaller than 0 when a participant systematically prefers bandits with smaller-than-average rewards.

#### Lagged regression

We next focused on learning, assessing how past task events affected participants’ future behaviour. Following a model-free approach, we used logistic regression to quantify the effects of past actions *a*_*t*−*i*_ and outcomes *r*_*t*−*i*_ on participant choices *a*_*t*_ and to compare the time courses of these effects between cognitive models (Fig. 4g). For each cognitive model, we calculated four regression models, one per bandit. There was no reason to respond differently to the four bandits, and indeed, the four regression models produced nearly identical results in all cases; hence, we averaged the results for visualization. Each regression model predicted the time course of choices for one particular bandit, *a*_1:__*n*_ (number of trials *n* = 150), coding trials as 1 when the bandit was chosen and 0 otherwise. We used two sets of regressors to predict *a*_1:__*n*_. ‘Bandit-reward’ regressors contain the time course of the number of points obtained in the past after choosing the current bandit: *r*_*i*:*n*+*i*_ × *a*_1:__*n*_. For example, the bandit-reward regressor at *t* − 1 contains the sequence of points obtained on the previous trial for those trials in which participants had chosen the current bandit; trials in which a different bandit was chosen contain the value 0. The second set of regressors are ‘other-reward’ regressors, which indicate the number of points obtained in the past after choosing a bandit other than the current bandit: *r*_*i*:*n*+*i*_ × (1 − *a*_1:__*n*_). We predicted choices *a*_1:__*n*_ from past events up to 20 trials in the past, 1 < *i* < 21, such that our models contained 40 regressors (20 bandit-reward and 20 other-reward regressors).

#### Mixed-effects regression

We next assessed how PC1 of participants’ reward state $\bf{s}_{t}^{(r)}$ (reward sensitivity) affected subsequent choices *a*_*t*__+1_ and response times *r*_*t*__+1_. To this aim, we ran a mixed-effects regression model specifying random effects of participants, including trial number and block number as nuisance predictors. For Fig. 3i, we preprocessed response times by log-transforming and then centring on the mean, individually for each participant and each block. We preprocessed PC1 of $\bf{s}_{t}^{(r)}$ by centring on the mean, individually for each participant and each block. Centring both measures across participants allows us to directly test for within-participant differences. This rules out the possibility that all observed differences arose from differences between participants, such that different participants occupied different states, which were also associated with differences in response times. Instead, the same participants transitioned through different regions of the space, which also captured differences in response times.

#### Multiple repeats and cyclic responses

We then focused on the structure within participants’ choice sequences. We calculated the average length of multiple repeats (continuous streaks that repeat the same action; Fig. 4d), and we counted the number of cyclic responses (four subsequent trials in which each of the four available actions is chosen once; Fig. 4e).

#### Compressibility ratio

We finally quantified the structure within participants’ choice sequences by estimating sequence compressibility (Fig. 4f). We used the Lempel–Ziv–Welch (LZW) algorithm, a relatively simple standard compression algorithm for sequential data^100,101^. LZW first identifies the subsequences (for example, ABCD or AAAA) that an original sequence is composed of and then re-expresses the original sequence in terms of these subsequences, hence reducing the sequence length by taking advantage of repetitions. Sequences that are composed of a small number of subsequences (for example, ABCDABCD) are more compressible than random sequences without such structure (for example, DADDCBDB). To estimate the compressibility of participants’ choice sequences, we first compressed each block’s original choice sequence using LZW, obtaining the compressed sequence length *l*_LZW_. For comparison, we also sampled random sequences of the same length as the original blocks (*n* = 150) using the same four elements (A, B, C and D). We also compressed these random sequences to obtain the baseline compressibility, *b*_LZW_, expected for sequences of the same length and with the same number of elements, just by chance. Finally, we calculated the ratio between the length of compressed random sequences and that of participants’ blocks, obtaining the compressibility score $\frac{{b}_{{\rm{LZW}}}}{{l}_{{\rm{LZW}}}}$.

### Model architectures

#### *Q*-learning model architectures

We obtained our Best RL model by comparing many variants of *Q*-learning^41^. In (tabular) *Q*-learning, each action *a* is associated with a value *Q*(*a*), which approximates the expected reward of *a* (ref. ^2^). Values are learned incrementally over trials, on the basis of the observed reward. On each trial *t*, the value of the chosen action is updated by a fraction *α* (called the ‘learning rate’) of the reward prediction error, the discrepancy between the reward *r*_*t*_ and the action value going into this trial, *Q*_*t*_(*a*):

1 $${Q}_{t+1}(a)={Q}_{t}(a)+\alpha \times ({r}_{t}-{Q}_{t}(a))$$

The standard formulation of *Q*-learning applies to environments with multiple states, where taking an action *a* in state **s** leads the agent to state **s**′. In such environments, the *Q*-value update includes a term corresponding to the *Q*-value of the subsequent state, including a discount factor *0* < *γ* < *1*. For example, the on-policy SARSA algorithm performs the following *Q*-value update:

$${Q}_{t+1}(\bf{s},a)={Q}_{t}(\bf{s},a)+\alpha \times ({r}_{t}+\gamma \times {Q}_{t}(\bf{s}^{{\prime} },{a}^{{\prime} })-{Q}_{t}(\bf{s},a))$$

In this paper, because the environment does not provide state transitions (for example, the subsequent state *s*′ does not depend on the previous state **s** and action *a*), we use a simplified algorithm without the term *γ × Q*_*t*_(**s**′, *a*′), following standard conventions in cognitive modelling^40,41^.

We compared our RL models head-to-head with neural networks. To make this comparison fair, we included a bias parameter *b* in the RL models. *b* allows a linear offset in value updates, a freedom that the neural-network models have by design:

2 $${Q}_{t+1}(a)={Q}_{t}(a)+\alpha \times ({r}_{t}-{Q}_{t}(a))+b$$

On any trial *t*, *Q*-learning agents select an action by transforming the vector **Q**_*t*_ of all four action values into a vector of choice probabilities **p**_*t*_ of the same length, using the softmax function. This transformation can have a ‘lower temperature’, leading to more deterministic choices by exaggerating differences between action values, or a ‘higher temperature’, leading to increasingly random choice. The inverse decision temperature *β* is a free parameter of the model:

3 $${{\bf{p}}}_{t}={\rm{softmax}}(\beta \times {{\bf{Q}}}_{t})$$

We call the model based on just equations (1) and (3) ‘Basic RL’. With only two free parameters (*α* and *β*), a Basic RL model typically does not predict human choices very accurately. Many extensions have been proposed to improve behavioural fit. We focus on three here: perseveration, forgetting and variable learning rates. Perseveration enables action repetition (or switching) independently of rewards and is the simplest form of reward-independent action-history processing. The perseveration term *c* adds a small bonus (of size *ϰ*) to the value of the action *a* that was chosen on the previous time step, but not to all other actions ⌐*a*:4

*Q*-learning agents that track both perseveration and action values have an additive choice rule. The vectors of action values and perseveration are added (to form ‘choice logits’ *h*_*t*_) and pass through the softmax rule for action selection:

$${{\bf{h}}}_{t}={{\bf{Q}}}_{t}+{{\bf{c}}}_{t}$$

$${{\bf{p}}}_{t}={\rm{softmax}}(\beta \times {{\bf{h}}}_{t})$$

Forgetting was implemented as the exponential decay of each action value back to *Q*_init_, at which each action value is initialized on the first trial. *Q*_init_ is a free model parameter that is fitted to participant behaviour. The decay parameter *f*, a free model parameter, determined the rate of decay. On each trial, all action values underwent forgetting, according to:

5 $${Q}_{t}(a)=(1-f)\times {Q}_{t}(a)+f\times {Q}_{{\rm{init}}}$$

Variable learning rates were implemented following a variant of the classic Pearce–Hall learning rule^102^, adapted to instrumental tasks^54^. In this model, each trial *t*’s learning rate *α*_*t*_ is updated on the basis of the previous trial’s reward prediction error *δ*_*t*_. The larger the absolute value of *δ*_*t*_, that is, the greater the ‘surprise’ about an outcome, the larger the learning rate:

6 $${\delta }_{t}={r}_{t}-{Q}_{t}(a)$$

7 $${Q}_{t+1}(a)={Q}_{t}(a)+{\alpha }_{t}\times {\delta }_{t}$$

8 $${\alpha }_{t+1}=w\times |{\delta }_{t}|+(1-w)\times {\alpha }_{t}$$

*w*, a free parameter of the model, is a weighting parameter that determines how variable (larger *w*) versus stable (smaller *w*) *α*_*t*_ is over time—a learning rate on the learning rate. At *w* = *0*, learning rates are stable, and the model reduces to simpler RL model variants. Variable-learning-rate model variants replace the standard learning rate parameter *α* with *α*_init_, the model’s initial learning rate on the first trial.

In the main text, we sometimes obliterate the subscript *t* in equations for better readability. Following common practice, we restricted the ranges of the free parameters of our *Q*-learning models to ensure interpretability. For example, a negative learning rate or negative forgetting would not be interpretable. We used common transforms (sigmoid, relu and tanh) to enforce the following ranges for RL models’ free parameters:

Learning rate / initial learning rate: 0 < *α* < 1, 0 < *α*_init_ < 1

Update bias: −1 < *b* < 1

Inverse decision temperature: 0 < *β* < ∞

Perseveration: −1 < *ϰ* < 1

Forgetting: 0 < *f* < 1

Weighting parameter: 0 < *w* < 1

The initial value *Q*_init_ was not restricted.

#### *Q*-learning model comparison

To identify the best *Q*-learning model for our data, we performed a systematic model comparison. We created 7^2^ − 1 = 48 model variants based on all parameter combinations. Supplementary Table 2 shows the results for the most relevant subset of model variants. Basic RL included only two parameters, *α* and *β*. Best RL included six parameters (*α*, *β*, *f*, *ϰ*, *b* and *Q*_init_). We fitted all models to the training split of our dataset, using the methods described in the following sections, and selected the winner on the basis of the model fit on the held-out test data.

#### RL-ANN architecture

RL-ANN has the same structure as Best RL but contains two neural networks instead of Best RL’s value update and perseveration operations (Fig. 2b). We first focus on the value update module, the model’s Reward ANN, and then turn to the perseveration network, the model’s Action-History ANN. The Reward ANN receives the same inputs as the classic value update (equation (1)), *Q*_*t*−1_(*a*) and *r*_*t*−1_, and produces the same output, *Q*_*t*_(*a*). On each trial *t*, the Reward ANN’s input layer vector $\bf{{i}}_{t}^{(r)}$ contains the concatenation of its two scalar inputs:

$$\bf{i}_{t}^{(a)}=[{Q}_{t-1}(a),{r}_{t-1}]$$

The activations in the hidden layer (the state vector $\bf{s}_{t}^{(r)}$) are obtained by passing the input vector through the first fully-connected layer of the network. Inputs are multiplied with the matrix of weights ${W}_{1}^{(\,r)}$, the bias vector $\bf{b}_{1}^{(r)}$ is added and the result is passed through a tanh nonlinearity:

$$\bf{s}_{t}^{(r)}=tanh\left({W}_{1}^{\,(r)}\bf{i}_{t}^{(r)}+\bf{b}_{1}^{(r)}\right)$$

The output of the network, *Q*_*t*_(*a*), is obtained by passing the state through a second fully connected layer, parameterized by weights ${W}_{2}^{\,(r)}$ and bias ${b}_{2}^{(r)}$ (there is no nonlinearity in the second layer; hence, values *Q* can be interpreted as logits):

9 $${Q}_{t}(a)={W}_{2}^{\,(r)}\bf{s}_{t}^{(r)}+{b}_{2}^{(r)}$$

Like Best RL, RL-ANN maintains a vector **Q**_*t*_ over trials, which contains one value per action. *Q*_*t*_(*a*) is replaced by the output of equation (9). All actions in **Q**_*t*_ undergo forgetting according to equation (8). The Reward ANN’s input layer has size 2 (containing *Q*_*t*−1_(*a*) and *r*_*t*−1_), and the output layer has size 1 (*Q*_*t*_(*a*)). The size of the hidden layer was determined by a hyperparameter sweep (see below).

RL-ANN’s Action-History ANN also is a three-layer, fully connected Multi-Layer Perceptron (MLP). The Action-History ANN receives the same input as classic perseveration (equation (4)), *a*_*t*−1_, and returns the same output, a vector **c**_*t*_ with one perseveration scalar per action. The network is parameterized by weight matrices ${W}_{1}^{\,(a)}$ and ${W}_{2}^{\,(a)}$, and biases $\bf{b}_{1}^{(a)}$ and $\bf{b}_{2}^{(a)}$:

$${i}^{(a)}t={a}_{t-1}$$

$$\bf{s}_{t}^{(a)}=tanh\left({W}_{1}^{\,(a)}\times {i}_{t}^{(a)}+\bf{b}_{1}^{(a)}\right)$$

$${{\bf{c}}}_{t}={W}_{2}^{\,(a)} \bf{s}_{t}^{(a)}+\bf{b}_{2}^{(a)}$$

The Action-History ANN’s input layer has size 1, and the output layer has size 4 (one per action). The size of the hidden layer was identical to the reward module’s hidden layer.

Like before, values **Q**_*t*_ and perseveration **c**_*t*_ are combined additively before passing through the softmax for action selection:

$${{\bf{h}}}_{t}={{\bf{Q}}}_{t}+{{\bf{c}}}_{t}$$

$${{\bf{p}}}_{t}={\rm{softmax}}({{\bf{h}}}_{t})$$

#### Context-ANN architecture

Context-ANN is an extension of RL-ANN that adds the ability to condition operations on the context (Fig. 3b). Context-ANN represents the reward context with the vector **Q**_*t*__−1_ and the action context with the vector **c**_*t*__−1_. We chose **Q**_*t*__−1_ and **c**_*t*__−1_ as context representations because they are the most succinct summaries of the past history and represent all four actions. Conditioning is performed by adding **Q**_*t*__−1_ and **c**_*t*__−1_ as inputs to the reward module and choice-MLP, respectively. In this way, the networks can learn to modify their operations on the basis of the additional context information (if this is supported by human behaviour):

$$\bf{i}_{t}^{(r)}=[{Q}_{t-1}(a),{r}_{t-1},{{\bf{Q}}}_{t-1}]$$

$$\bf{i}_{t}^{(a)}=[{a}_{t-1},{{\bf{c}}}_{t-1}]$$

Everything else remains the same as in RL-ANN (see above).

#### Memory-ANN architecture

Memory-ANN is our winning model. It is an extension of Context-ANN that allows a more flexible context representation. Instead of conditioning on the output vectors **Q**_*t*__−1_ and **c**_*t*__−1_, Memory-ANN conditions on their precursors, the hidden states $\bf{s}_{t-1}^{(r)}$ and $\bf{s}_{t-1}^{(a)}$. As a simplification, it removes the dependence on *Q*_*t*__−1_(*a*):

$$\bf{i}_{t}^{(r)}=[{r}_{t-1},\bf{s}_{t-1}^{(r)}]$$

The remaining processing steps are unchanged:

$$\bf{s}_{t}^{(r)}=\tanh \left({W}_{1}^{\,(r)}\bf{i}_{t}^{(r)}+\bf{b}_{1}^{(r)}\right)$$

$${Q}_{t}(a)={W}_{2}^{\,(r)}\bf{s}_{t}^{(r)}+{b}_{2}^{(r)}$$

#### Vanilla RNN model architecture

Vanilla RNN is a basic RNN. On each trial *t*, the model receives information about the most recent action *a*_*t*__−1_ and the reward received after choosing this action, *r*_*t*__−1_, and returns a vector of choice logits **h**_*t*_, with one element for each action. Like before, choice logits guide the selection of the next action *a*_*t*_, after transformation into action probabilities using the softmax function:

$${{\bf{p}}}_{t}={\rm{softmax}}({{\bf{h}}}_{t})$$

Vanilla RNN is a simple, fully connected, recurrent three-layer network. It concatenates the inputs **a**_*t*__−1_ (a one-hot vector indicating the chosen action with 1 and all others with 0) and *r*_*t*__−1_ (a scalar) into a joint vector **i**_*t*_, the input activations of the network:

$${{\bf{i}}}_{t}=[{{\bf{a}}}_{t-1},{r}_{t-1}]$$

The hidden layer (or recurrent state **s**_*t*_) is obtained by passing the input activations through the first layer of fully connected neurons, parameterized by weight matrix *W*_1_ and biases *b*_1_, in the same way as above:

$$\bf{s}_{t}=\tanh ({W}_{1}\bf{i}_{t}+\bf{b}_{1})$$

The final output, the vector of logits **h**_*t*_, is the result of passing the state through another fully connected layer, parameterized by weight matrix *W*_2_ and biases *b*_2_:

$${{\bf{h}}}_{t}={W}_{2}\bf{s}_{t}+\bf{b}_{2}$$

Action choices are made like before, by passing choice logits through a softmax function to determine choice probabilities:

$${{\bf{p}}}_{t}={\rm{softmax}}({{\bf{h}}}_{t})$$

### Model training

#### Data splits

We randomly split our dataset into three partitions: training (80% (690) of participants; 3,302 blocks), testing (10% (86) of participants; 413 blocks) and validation (10% (86) of participants; 419 blocks). We used the same train–validation–test splits for testing all models. In other words, the same exact sessions went into the training split for each model, a different set of sessions went into the testing set for each model and a third set was used for validation of all models. We did this to ensure that the resulting model fits were comparable between models.

The training data were used to fit the model parameters (for example, *α*, *β*, *W*_1_ and *b*_2_) of a wide range of models, including all combinations of all hyperparameters (for example, the number of hidden units; see below). The validation data were used to identify the optimal set of hyperparameters for each model. The test data were used to determine the fit of each selected model (Figs. 2d and 3c). The three-way split was necessary for two reasons. The validation split allowed us to find the best hyperparameters for each model. This ensured that differences in model fits reflected differences between model architectures rather than differences in the optimality of the chosen hyperparameters. For example, we can be sure that no Context-ANN—whatever its hyperparameters—could ever beat Memory-ANN, because there is no Context-ANN that fits the data better than the one we report. The test split was necessary to ensure that models did not overfit to the training data.

#### Model fitting

All models, both classic variants of *Q*-learning and neural networks, were trained with the Adam optimizer, using the optax package (https://github.com/google-deepmind/optax) for jax (https://github.com/google/jax). The optimizer learning rate, batch size, number of training steps, weight decay and number of hidden units (if applicable) for each model were determined by a hyperparameter sweep. Each training batch was sampled randomly and with replacement from the training data. We systematically assessed the following space of hyperparameters: learning rate, 1 × 10^−3^, 1 × 10^−4^, 1 × 10^−5^; L2 weight decay, 1 × 10^−3^, 1 × 10^−4^, 1 × 10^−5^; number of the hidden units, 16, 32, 64; batch size, 32, 64, 128. We trained each model for 1,000,000 steps on the training data, using five instantiations of each combination of hyperparameters, and identified the number of training steps (≤1,000,000) and hyperparameters that led to the best fit on the validation data. The chosen hyperparameters for each model are shown in Supplementary Table 1.

#### Fitting objective

The goal of training was to create models that behave as similarly as possible to humans (rather than to perform the task as well as possible). We followed standard practices^41^ to achieve this. We minimized the negative log-likelihood loss (also called cross-entropy) of each model with respect to the training data. This loss incentivizes model parameters that maximize the (log) probability of jointly predicting the choices *a*_{*t*,*i*}_ of each participant *p* on each trial *t* in a training batch (of size bs), by following stochastic gradient descent over training batches:

$$L=-\mathop{\sum }\limits_{i=1}^{\mathrm{bs}}\mathop{\sum }\limits_{t=1}^{{n}_{\mathrm{trials}}}log(p({a}_{t,i}))$$

The optimal batch size bs was determined individually for each model on the basis of a hyperparameter sweep (see above). Each task had *n*_trials_ = 150.

To obtain the final fit for each model (Figs. 2d and 3c), we calculated the loss of the variant with the best hyperparameters on the held-out test data. We calculated the loss separately for each task block, so that we could assess the variability between participants. We also transformed model losses into the trial-wise prediction accuracy, an estimate of what percentage of human choices are predicted accurately:

$$\mathrm{acc}=\mathrm{exp}\left(\frac{-L}{bs\times {n}_{trials}}\right)$$

### Model analysis

#### Qualitative model fit

We created a synthetic dataset for each model, using the hyperparameters (for example, batch size; Supplementary Table 1) and parameters (for example, learning rate *α* and connection weights *W*_1_) we obtained in model fitting. We simulated behaviour on the same 4,134 tasks (with the same reward schedules) as human participants, using ‘open-loop’ simulation (which means that human choices are unknown to the behaving models). We then subjected human and model behaviour to the same statistical analyses to uncover qualitative similarities and differences (Fig. 4).

#### Model dynamics

We also created ‘closed-loop’ simulations for each model. Also called ‘teacher forcing’, this means that a model is forced to make the same choices as a participant. The model does not sample its action from the action probabilities it calculates on each trial but instead automatically selects the teacher’s choice. We used this method to inspect the internal dynamics (for example, trial-by-trial trajectories of values *Q* and choice kernel *c* or memory states **s**) that our models assigned to individual participants (Supplementary Fig. 5).

#### Model inspection

The reward module (described above) determines how observed rewards *r*_*t*__−1_ map onto values *Q*_*t*_. We analysed this mapping by probing reward modules with the full range of inputs and measuring their output (Fig. 2e). We first extracted the relevant parameters (${W}_{1}^{(r)}$, ${W}_{2}^{(r)}$, $\bf{b}_{1}^{(r)}$ and $\bf{b}_{2}^{(r)}$) from the fitted model (RL-ANN or Memory-ANN). We then initialized a new MLP with the same shape as the original reward module (for example, for Memory-ANN: 2 input units, 32 hidden units and 1 output unit) and injected the fitted parameters. We uniformly sampled rewards *r*_*t*__−1_ between 1 and 100 points. For RL-ANN, we also sampled values *Q*_*t*__−1_(*a*) between the 10% and 90% quantiles of the values observed in the closed-loop dataset. For Memory-ANN, we sampled hidden state vectors $\bf{s}_{t-1}^{(r)}$ along the first (or a different) principal component of the hidden states visited in the closed-loop data; samples were taken up to 1.5 standard deviations from the mean. We finally collected the outputs *Q*_*t*_(*a*) of this MLP in response to each combination of inputs.

The same method was used to analyse the action-history module. We obtained the corresponding fitted parameters (${W}_{1}^{(a)}$, ${W}_{2}^{(a)}$, $\bf{b}_{1}^{(a)}$ and $\bf{b}_{2}^{(a)}$) and injected them into a newly initialized MLP. We sampled actions *a* uniformly; for Memory-ANN, we also sampled hidden state vectors $\bf{s}_{t-1}^{(r)}$, using the same method as above. We then collected the output $\bf{c}_{t}^{(a)}$ of the network and visualized the relationship between inputs and outputs (Supplementary Fig. 6).

To assess the contents of $\bf{s}_{t}^{(r)}$ (Fig. 3j,k), we calculated a separate regression model for each delay *i*, predicting the reward observed on trial *t* − *i* on the basis of a PC of the current state $\bf{s}_{t}^{(r)}$. We repeated this analysis individually for each PC.

### Reporting summary

Further information on research design is available in the Nature Portfolio Reporting Summary linked to this article.

## Supplementary information

Supplementary Information Supplementary Tables 1–9, Figs. 1–12, Results and Discussion.

Reporting Summary

## Data Availability

The dataset generated for this study is available via the Open Science Framework at https://osf.io/8xz3w/.

## Code availability

The code generated for this study is available via GitHub at https://github.com/google-deepmind/hybrid_rnns_reward_learning. Please refer to the Colab provided for a simple demo of how to fit the models presented here.
