# Supplementary material for: Hybrid neural–cognitive models reveal how memory shapes human reward learning
Source: Nat Hum Behav. 2026 Feb 5;10(5):972–87. doi: 10.1038/s41562-025-02324-0 (PMC13212159; doi:10.1038/s41562-025-02324-0)
Supplement: Supplementary file 1 — Supplementary Tables 1–9, Figs. 1–12, Results and Discussion. [file 41562_2025_2324_MOESM1_ESM.pdf]

nature human behaviour

Supplementary information https://doi.org/10.1038/s41562-025-02324-0

Hybrid neural–cognitive models reveal how memory shapes human reward learning

In the format provided by the authors and unedited

Supplemental Material

Contents

Supplementary Results **2**

Fitted Parameters of the Best RL Model 2

Testing Model Recovery and Function Recovery in Simulation 2

Robustness Checks 5

Variable Learning Rate Extensions for RL 7

Individual Differences 11

Latent Dynamics 16

Action Processing Channel 20

Effect of Training Data Size and Other Hyperparameters 23

Additional PCA Results 27

Additional Model Fits 27

Additional Qualitative Model Analyses 31

Causal Manipulation on Latent Model States 35

Supplementary Discussion **44**

Table 1: Chosen hyperparameters for the main models. "Steps": Number of training steps. "Opt. Learn. Rate:" Learning rate for the Adam optimizer. "Units:" Number of units in the hidden layer. "L2 Weight:" Weight of the regularization term on the L2 norm of model parameters. "Batch Size:" Number of task blocks per training batch. "N Params.": Resulting number of free parameters for each model that are fitted during training on human behavior.

Model	Steps	Opt. Learn. Rate	Units	L2 Weight	Batch Size	N Params.
Best RL	300k	1e-4	NA	1e-5	32	6
RL-ANN	1M	1e-4	16	1e-5	32	216
Context-ANN	1M	1e-4	64	1e-5	64	1,352
Memory-ANN	1M	1e-4	32	1e-5	128	2,472
Vanilla RNN	1M	1e-4	64	1e-5	64	4,740

Supplementary Results

Fitted Parameters of the Best RL Model

Fitting Best RL to human participants revealed the following parameter values: inital learning rate $\alpha_{init} = 0.46$, forgetting rate $f = 0.046$, variable learning rate parameter $w = -0.012$, perseveration $\kappa = 0.071$, bias $b = -0.171$, and inverse decision temperature $\beta = 9.21$. As expected, this model fit the data substantially worse than Vanilla RNN: it only predicted an average of 60.6% of participant choices in the held-out test data (versus 68.3% for Vanilla RNN; $p < 0.0001$, $t(412) = 28.9$). Hence, the Best RL model fell significantly short in predicting human behavior, showing that standard RL models do not adequately describe human reward learning on our task.

Testing Model Recovery and Function Recovery in Simulation

We wanted to ensure that our method can reliably identify learning algorithms from observed behavior. To do this, we tested the method on simulated data, where we had access to the ground-truth models and their operations. We simulated artificial behavior from each of our four main models (Best RL, RL-ANN, Context-ANN, Memory-ANN, Vanilla RNN), based on

the parameters we had obtained when fitting the human dataset. We used the same number of participants and the same reward schedules as in our human sample (refer to “open-loop simulation” above). We then fitted a fresh version of each model to the training split of each simulated dataset, and calculated the loss on its held-out test split. We used the following hyperparameters for all models: 1M training steps; optimizer learning rate: 1e-4; batch size: 32; number of hidden units: 16; L2 weight decay: 1e-5. We did not perform hyperparameter sweeps due to prohibitive resource demands. However, based on our experience with the chosen hyperparameters, we do not expect the results to change qualitatively after a sweep.

We observed that all five models achieved similar out-of-sample prediction for the simplest model, Best RL (cross-entropy loss between 93-94; bottom row, Fig. S1). This suggests that all models were able to explain the behavior similarly well, and even the most flexible neural network models did not overfit. To select a single winning model, we employed a simplicity prior, which favors the selection of the simplest model that achieves the best fit. This approach led us to correctly identify Best RL as the model that produced the dataset. The subsequent models showed the same pattern: All models besides Best RL captured the behavior of RL-ANN agents equally well (loss of 85 versus 81-82; Fig. S1, second row from the bottom). A simplicity bias hence correctly identifies RL-ANN as the underlying model. The same was true for Context-ANN, which was not captured as well by Best RL and RL-ANN, but equally well by Context-ANN and Memory-ANN, such that Context-ANN could be correctly selected. And the same approach also worked for the final model, Memory-ANN. However, Memory-ANN and Vanilla RNN were not identifiable on the given task. The reason is simple: if both models are indeed able to perfectly imitate humans, then they should not be recognizable based on their simulated behavior. (And because Memory-ANN is a simpler model, and hence more data efficient, it might not be surprising that it even fit Vanilla RNN behavior better.) However, this lack of model identifiability on the current task does not imply that Memory-ANN and

Vanilla RNN are equally expressive in general. We provide one example task, in which models would show different behavior, making them identifiable: Memory-ANN cannot learn about interactions between rewards and action sequences, due to its separation of modules. A simple task that Vanilla RNN could learn, but Memory-ANN cannot, would hence be a situation in which the outcomes are not valued the same for all actions (inducing interactions between actions and outcomes). Imagine a simple task in which points count negative for the 'blue' action but positive for all others. Vanilla RNN would be able to learn the task, but Memory-ANN cannot. In sum, we were able to select the correct model for each dataset, confirming that our models (except Vanilla RNN) can be identified based on observed behavior and that they are distinct from each and identifiable.

We also wanted to confirm that we could accurately distill cognitive operations. We tested this on the two diametrically opposed models, Best RL and Memory-ANN. While Best RL employs a simple fixed Q-value update, Memory-ANN employs an intricate algorithm that combines fast and slow learning (Fig. 3E and F). We wanted to ensure that our method worked for both extremes. We hence simulated behavior from both models and verified that the ground-truth algorithm was accurately recovered. We used an RL-ANN model to infer the learning algorithm employed by Best RL, and a Memory-ANN model to infer the learning algorithm employed by the Memory-ANN version that was trained on human behavior. We used the same behavioral datasets and the same fitted models as for Fig. S1A (Best RL / RL-ANN pair: bottom row, second-to-left; Memory-ANN / Memory-ANN pair: top right). To visualize the fitted functions, we used the same methods as explained above (section "Model Inspection"). Both cases led to very good recovery (Fig. S1B): RL-ANN's reward module recovered Best-RL's Q-learning update almost perfectly. Fig. S1B (top) shows the ground-truth on the left (the function of Best RL used in simulation), and the recovered mapping on right (reward module extracted from fitted RL-ANN). In truth, RL-ANN recovers the linear mapping from rewards

r_t to value $Q_{t+1}(a)$ with a linear grading according to $Q_t(a)$. However, the ground-truth and the recovered version differ in terms of their slope. This difference arises because of a small structural difference between Best RL and RL-ANN: Best RL has an inverse decision temperature parameter β that multiplies q-values before passing them through the softmax (simulated $\beta = 8.4$). RL-ANN does not have this parameter, which means that it needs to scale values differently, namely during value calculation within the reward module. For this reason, the output of Best RL's value update function are unscaled Q-values (between 0-1), whereas the output of RL-ANN's reward module are already-scaled values (potentially larger than 1 if $\beta > 1$). (For aficionados: The mapping between β and slope is not perfect because the forgetting parameter κ operates before scaling by β in Best RL, but after scaling in RL-ANN. We initially included a parameter β in the RL-ANN model, but found that it was difficult to recover and led to trade-offs with other parameters in the model. For this reason, we removed β in the final version presented in this study.)

We also found that we could excellently recover the complex algorithm by the fitted Memory-ANN processed rewards. We inspected the fitted model's reward module, using the same methods as before (Fig. S1B, middle and bottom row). We found that the input r_t and the output Q_{t+1} showed the same sigmoid-like relationship as the ground-truth, with a similar modulation of steepness by the first principal component of the latent state $s_t^{(r)}$ (Fig. S1B, middle row). The latent state was also recovered very well: in the fitted model, just like in the ground-truth simulation, "reward sensitivity" (PC1 of $s^{(r)}$) gradually increases after receiving larger rewards (Fig. S1B, bottom row).

In sum, our method allows us to accurately recover both the architecture (Fig. S1A), and the operations of models for which we know the ground-truth (Fig. S1B). We hence have reasonable confidence that the same method, applied to humans, can shed light on the cognitive architecture as well as on the cognitive operations performed.

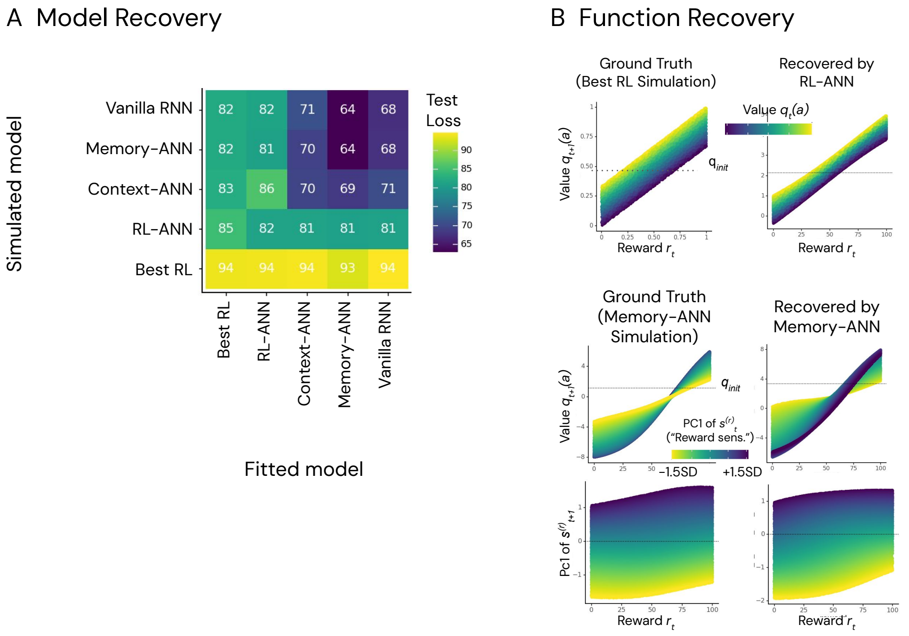

Figure 1: A) Model Recovery. We simulated behavior from each of our four main models (y-axis) and then fitted each dataset using the same methods as in the main study (x-axis). The matrix colors show the loss (cross-entropy / negative log likelihood) that each fitted model achieved on the held-out test split of each dataset. Smaller numbers (darker colors) indicate better fit. We show the precise fits of each model (rounded to the closest integer) using white numbers. B) Function Recovery. The left column shows the ground-truth operations of the simulated model, which we obtained by fitting free model parameters to human behavior (top: Best RL; middle and bottom: Memory-ANN). The right column shows the corresponding functions extracted from the models we fitted to the stimulated datasets (top: RL-ANN; middle and bottom: Memory-ANN). Both the fixed Q-value update (top) and the intricate interplay between quick value updating (middle) and slow states (bottom) were recovered very well.

Robustness Checks

We also assessed the robustness of our results. We wanted to make sure that we did not report incidental patterns that arose just once in a single model, but only results that consistently replicated across variations. To this aim, we repeated our analyses on a second, fresh version of Memory-ANN that was fitted independently of the first, and used a different random seed (but the same hyperparameters; see above). Model fits were identical to the first decimal and Fig. S2 shows that the results regarding cognitive operations hold up as well. The new model extracts very similar algorithms both for the quick learning component (mapping rewards r_t to values Q_{t+1}; Fig. S2A, top), and for the slow state component (mapping previous states $s_t^{(r)}$ to new states $s_{t+1}^{(r)}$, based on the observed reward r_t; Fig. S2A, bottom). The new model also shows a similar combination of long-term (Fig. S2B, top) and quick state components (Fig. S2B, bottom). Even the ordering and identity of the principal components was very similar (with the exception of PC8). The similarity between these two models gives an indication of how robust our findings are. While small differences exist, the overall patterns are likely reliable.

Variable Learning Rate Extensions for RL

Given that the main advantage of Memory-ANN over Best RL is the gradual adjustment of updating mechanisms based on the history of past outcomes, it is possible that a simpler variable learning rate model could do the same. To test whether variable learning rate models can indeed explain human behavior better than Best RL (and potentially as well as Memory-ANN), we tested several new model variants. Each one extends Best RL by allowing for a different kind of variable learning rate.

In particular, we test a set of models based on one introduced by Li et al. (2011), in turn drawing on classic work by Pearce and Hall (1980). The core idea is that the learning rate is itself varying from trial to trial, such that learning slows down when prediction error magnitudes

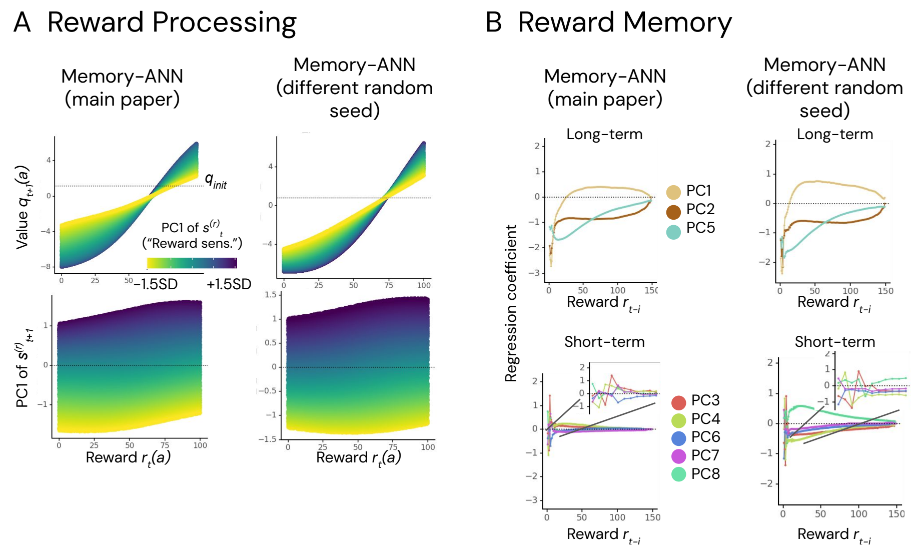

Figure 2: Robustness Check. A) Recovered Update Function. The left column shows the results of the main model (copied from main Fig. 3F). The right column shows the results of a new model, fitted independently and based on a different random seed. For details, see main text and original figure legend. Both models show very similar results. B) Recovered Memory Components. Left and right column like before. See main Fig. 3J-K and main text for details. Both models again lead to very similar results.

are lower (and learning is unnecessary) and speeds up when prediction errors are higher. The core model keeps track of a running average of absolute prediction errors, and we then consider a few different variants that transform this quantity monotonically into a dynamic learning rate time series. In particular, on each trial t, the current learning rate α_t is modified based on the previous trial's reward prediction error δ_t. The larger the absolute value of δ_t, that is, the greater the "surprise" about the last trial's outcome, the more the learning rate will increase on the subsequent trial $t + 1$:

$$\delta_t = r_t - Q_t(a)$$

$$Q_{t+1}(a) = Q_t(a) + \alpha_t * \delta_t$$

$$\alpha_{t+1} = w * |\delta_t| + (1 - w) * \alpha_t$$

w, an additional free parameter of the model, is a weighting parameter that determines how variable (larger w) versus stable α_t is over time (smaller w). At $w = 0$, learning rates are stable and the model reduces to Best RL. At $w > 0$, w acts like a learning rate on the learning rate α. The new model also includes the additional free parameter $0 < \alpha_{t=0} < 1$, the model's initial learning rate on the first trial. Hence, our first variable learning model has eight free parameters $(\alpha, \beta, \kappa, p, Q_{init}, b, \alpha_{t=0}, w)$. We refer to this model as the "simple variable learning rate model".

We fitted this model to the main text dataset, achieving a prediction accuracy on the held-out test data of 60.5%. For reference, simple Best RL achieved an accuracy of 59.9% (paired t-test: t(412)=10.01, $p < 0.0001$) and Memory-ANN achieved 68.3% (paired t-test: t(412)=27.82, $p < 0.0001$; see Figure S3). This shows that adding variable learning rates to our Best RL

model indeed improved behavioral predictions; however, the new model was far from reaching the same level of prediction as Memory-ANN.

It is possible, however, that the variable learning rate model introduced above simply is too rigid to capture participant behavior, but that a slight increase in flexibility would allow it to capture human behavior. To test this, we implemented a second variant that added a flexible, nonlinear transformation of the learning rate based on additional learned parameters. We refer to this models as the "persistent nonlinear variable learning rate model":

$$\delta_t = r_t - Q_t(a_t)$$

Applying a non-linear transformation to learning rate α_t procures the new variable α'_t, which is then used going forward:

$$\alpha'_t = tanh(b_0 + b_1 * \alpha_t)$$

$$Q_{t+1}(a_t) = Q_t(a_t) + \alpha'_t * \delta_t$$

$$\alpha_{t+1} = w * |\delta_t| + (1 - w) * \alpha'_t$$

This model includes two additional free parameters, $-\infty < b_0 < \infty$, the intercept of the transformation, and $-\infty < b_1 < \infty$, its slope, for a total of 10 free parameters. The model achieved an accuracy on the test data of 61.1%, which improves further upon the first variable learning model (paired t-test: t(412)=7.94, $p < 0.0001$), but does not reach Memory-ANN's performance (68.3%).

This second model assumes that the nonlinear transformation applied to the learning rate persists over time. However, it is possible that a better fit could be achieved if the transformation only applied to the current trial. To test for this possibility, we created a third model in which

only the trial-by-trial updates persist over time, but the nonlinear transformation is temporary. We refer to this models as the “nonlinear variable learning rate model”:

$$\delta_t = r_t - Q_t(a)$$

Applying a non-linear transformation to learning rate α (just for the current trial):

$${}_{used} = tanh(b_0 + b_1 * \alpha_t)$$

$$Q_{t+1}(a) = Q_t(a) + \alpha_{used} * \delta_t$$

$$\alpha_{t+1} = w * |\delta_t| + (1 - w) * \alpha_t$$

This model achieved a slightly better prediction accuracy of 61.2% on the testing data compared to the persistent variable learning rate model (paired t-test: t(412)=4.02, $p < 0.0001$), even though the overall achievement is still far below Memory-ANN.

Taken together, these results indicate that hand-crafted variable learning rate models of this kind—including highly-flexible adaptations—do indeed improve behavioral predictions compared to Best RL, but fall far below the fit of Memory-ANN. This indicates that variable learning rate models of this kind are not comparable to hybrid models as an explanation for human behavior in our task.

Individual Differences

There is no agreed-upon way to model individual differences when using neural networks, and while some previous studies have resorted to fitting each individual with a separate model (e.g., Ji-An et al., 2024; K. Miller et al., 2023), others have explicitly included axes of individual variation within meta-models that are fitted to the entire population (e.g., Dezfouli et al., 2019;

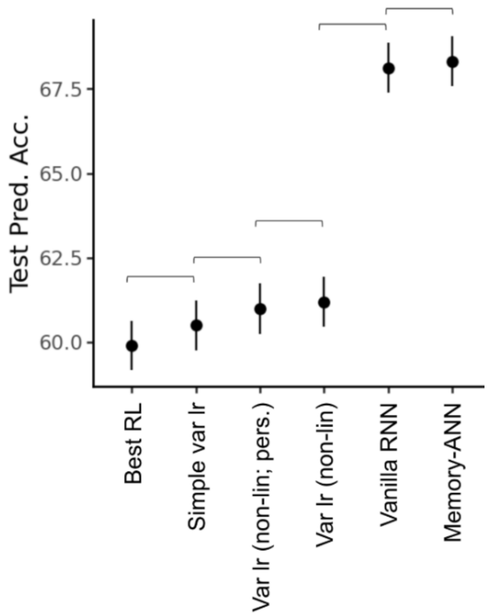

Figure 3: Fit (prediction accuracy) of models presented in the main text and variable learning rate models.

Song et al., 2021), and yet others have not attempted to explicitly model individual differences, and instead relied on just one meta-model that is fitted to all participants, like in our study (e.g., Peterson et al., 2021). It is not clear how our main findings—namely the results of the model comparison and our conclusions about the cognitive mechanism—depend on this choice. This issue can be addressed from two angles: 1) Relying on additional analyses that directly compare individually-fitted models to "meta-models" that are fitted to the population. 2) Dissecting models' latent states to differentiate within-participant and between-participant differences.

The first main finding of our study is that the Memory-ANN model fits participant behavior better than the Best RL model. We interpret this finding as suggesting that Memory-ANN better matches the human cognitive process than Best RL. However, it is possible that instead, the difference in model fit arises because the models deal differently with individual differences. Indeed, Memory-ANN's latent state gives it the ability to both store rich information about in-

dividuals' action and reward histories, and to potentially derive and retain latent variables that describe individual differences. Hence, Memory-ANN can adapt "in context" to individuals' behavior even though the learned parameters of the model are fixed. This ability to change behavior despite frozen model weights is related to meta-learning, where an agent with frozen weights quickly adapts to a new task (e.g., Wang et al., 2018) and to in-context learning in LLMs (e.g., Lampinen et al., 2024). Best RL, on the other hand, does not have the ability to adjust to individual differences once the model parameters are frozen. These differences are the focus of a recent simulation study that compares linear and non-linear models in their ability to capture individual differences (Katahira, 2024). The feature that allows ANN-based models to adjust to individual differences even with frozen weights is termed "individual difference tracking" (IDT). In sum, it is possible that in our study, Memory-ANN fitted participant behavior better than Best RL simply because it better captured individual differences, and not because its underlying cognitive algorithm is more accurate.

We addressed this issue by equating both models' ability to capture individual differences, fitting Best RL to individual participants instead of the whole population. To do this, we first had to split our dataset differently. Instead of assigning some participants to the training split and other participants to the (validation and) testing split, we split the dataset within participants in order to make held-out predictions about individuals. We assigned $n - 1$ sessions of each participant to the training data and the remaining n_{th} session to the testing data. We repeated this process for each session 1-5 to obtain five leave-one-session-out data folds.

Because the new training dataset differed from the one in the main paper (e.g., smaller size of each training split), we first re-fit the original models to obtain adequate baselines. For Best RL, a single set of parameters ($\alpha, \beta, \kappa, p, Q_{init}, b$) was fitted to the entire training data, just like before. We then assessed the model's accuracy in predicting the behavior in the testing data, using this single set of parameters. We averaged the prediction accuracy across the five data

folds and across all participants, obtaining a performance of 59.9% (see Fig. S4). We also refitted Memory-ANN to the newly-split dataset, using the same hyperparameters as before. Memory-ANN achieved an average prediction accuracy on the testing data of 68.6%. (Note that even though Memory-ANN was fitted to the entire population, the model's flexibility with in-context adaptation presumably allows it to capture individual differences, unlike Best RL with a single set of parameters shared across subjects.)

Finally, we calculated the cross-validated fit of the new variant, an individually-fitted version of Best RL. We used maximum likelihood estimation (MLE) to identify one set of parameters ($\alpha, \beta, \kappa, p, Q_{init}, b$) for each participant, fitting the model parameters to each participant's $n-1$ training sessions, and calculating the prediction accuracy of these parameters for the same participant's n_{th} held-out session. The average score over participants and data folds was 61.1%, a small but significant improvement over the population-based Best RL (paired t-test: t(412)=6.61, $p < 0.0001$). However, this model still fell far short of the newly fitted Memory-ANN (t(412)=30.03, $p < 0.0001$), demonstrating that equating the ability to capture individual differences did not eliminate Memory-ANN's advantage over Best RL as an architecture. This supports the conclusion that the cognitive algorithm represented by Memory-ANN is a better match to human participants than that represented by Best RL, and verifies that the result was not simply due to the ANN's ability to capture individual differences more nimbly.

The second key aspect of our results is a set of analyses aimed at unpacking the sources underlying Memory-ANN's fit advantage, interpreted as revealing aspects of the within-participant mechanisms of learning. This includes, for instance, results on the relative advantage of different RNN architectures, and the analyses of the best-fit network's latent state dynamics. For concreteness, we focus on the latter results here. In principle, given that Memory-ANN's fit to data comprises both in-context adjustment to different participants' individual differences, and within-participant learning, the properties of the latent state we discover (such that it reflects

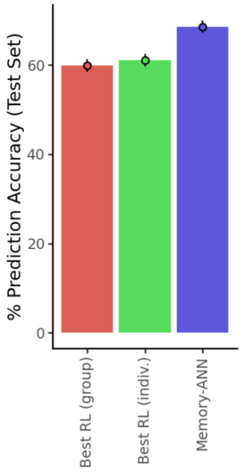

Figure 4: Fits (prediction accuracy on the held-out cross-validation test splits) of Best RL (individually fitted / fitted to the entire population) and Memory-ANN models.

history at multiple timescales) might reflect either between- or within-participant effects.

Thus, to rule out the possibility that these aspects of network latent state solely reflect adjustment to participants' individual styles, and demonstrate that they instead at least in part capture within-participant progression of learning over the experiment, we analyzed Memory-ANN's latent activations, finding evidence that the model successfully captured such within-participant changes in strategy.

Specifically, we ruled out that individual differences were the only source of the previously analyzed latent state dynamics. We hence aimed to show that at least some latent activity captured differences within participants. To this end, we performed a PCA on Memory-ANN's latent state (see main paper and supplemental methods above for details) and analyzed how the underlying PCs covaried with individual participants' observed behavior, while controlling for between-participant differences. We found that changes in the PCs indeed predicted differences within participants (see below). For example, controlling for each participant's mean noisiness, participants showed relatively noisier responses on trials when their values on PC1

were low (yellower curves) than while they were high (see Fig. 3K in the main text). Similar within-participant changes were evident for response times (see Fig. 3L in the main text), relative to each participant's mean. These results show that Memory-ANN's latent state activations successfully captured behavioral changes within the same participant, suggesting that Memory-ANN captured how participants gradually adjusted their behavior over time, rather than gradually adjusting to individual differences. These results accord with our interpretation that individuals gradually update their latent memory states while performing the task, which results in a continuous adjustment of their observed behavior strategies. Memory-ANN's latent state dynamics hence likely capture the cognitive mechanism of gradual adjustment, rather than exclusively reflecting IDT.

In sum, we conclude that both main aspects of our findings—the results of the model comparison and our interpretation of the winning model—do not change when we take individual differences into account.

Latent Dynamics

There are several features that make computational models interpretable. We first focused on latent processes. It has been argued that access to latent processes allows insight into an agent's cognitive process as it unfolds over time. Our winning model, Memory-ANN, exposes several latent processes besides trial-by-trial reward processing. Choice logits $h_t(a)$ (Fig. S5C) determine (stochastically) which action a is chosen on each trial t, and are the most direct predictor of human choice (Fig. S5B). Possibly, this latent variable exposes participants' final valuation of their available options, and might provide insight into the neural substrates underlying choice.

This final choice logits h (dropping a and t for readability) is composed of two separate parts, which do not interact with each other. Q is based solely on the history of rewards, whereas c only has access to a participant's own history of chosen actions. Because they rely on distinct

sources of information, both parts show strikingly different time courses (compare Fig. S5D versus E). In future work, this distinction could help dissociate the neural systems that underlie reward learning from habitual choice and perseveration by employing Q and c as neural regressors.

Another way in which Memory-ANN goes beyond many previous cognitive models is in that it explicitly models the complex, high-dimensional cognitive states that provide the foundation for later processing. Both reward-based and action-based processing systems maintain these high-dimensional recurrent states, which follow their own intricate dynamical patterns ($s^{(r)}$ and $s^{(a)}$, respectively). Using these time courses as neural regressors might make it possible to investigate the complex, but foundational cognitive states that are at the heart of decision making but have so far been out of reach. In the main text, we focused exclusively on the first principal component (PC) of the reward system's hidden state ("reward sensitivity"). Fig. S5F shows the time courses of three more PCs in the reward system, some of which follow fast trajectories (e.g., PC3), while others change more gradually (e.g., PC1, PC2, PC4; for explained variance, see Fig. S5I). Provocatively, some PCs show systematic changes over time, which persist across participants (Fig. S5G, bottom). For example, "reward sensitivity" (PC1) rises, which might indicate changes in participants' processing of rewards, and a transition from a more exploratory strategy early on to a more exploitative strategy as the task progresses. Components of the latent state $s^{(a)}$ of the action-based processing channel are shown in Fig. S5G. Again, these patterns were qualitatively different from those of the reward-based channel (Fig. S5F). Both participants' individual trajectories (top) and average trajectories (bottom) showed pronounced and unique patterns, suggesting that it might be possible to dissociate the underlying neural systems.

How do Q-values differ in Memory-ANN compared to standard RL? In RL, Q-values are defined so as to approximate expected rewards, but there is no theoretical definition for their

counterpart in Memory-ANN—here, Q-values are just latent variables that provide a convenient explanation for human behavior. To determine whether these variables reflect reward magnitudes, we calculated the correlation between Q_t and c_t (before choosing an action) and the reward r_t received after choosing the action (Fig. S5H). h and Q were significantly correlated with rewards, showing small to medium effect sizes (h: $r = 0.13$; Q: $r = 0.21$; both $p < 0.0001$). c, on the other hand, showed a negligible relationship ($r = 0.0017$, $p = 0.0069$). This shows that choice logits were related to reward magnitudes, even though the mapping is not perfect, especially for low reward magnitudes.

Figure 5 legend, continued: D) Values Q. Q is one of the two components of h, calculated by Memory-ANN's "reward module" (see "Model Architectures"). Individual time courses (top) show the combination of reward-based learning with forgetting: $Q(a)$ increases steeply after a large reward, while $Q(\neg a)$ decays gradually toward the initial values Q_{init}. Averages over participants (bottom) are not biased towards any action. Changes over time can be interpreted in this case because they lead to a shift in the balance between reward module and action-history module: if reward module contributes larger values to h on one trial, the weight of action-history module on this trial is automatically smaller. Such changes can lead to a shift in control between the two systems over time. E) Choice kernel c. The second component of h is produced by "action-history module". Individual traces (top) reveal participants' strong propensity to repeat choices: the choice kernel $c(a)$ keeps increasing as long as action a is chosen, while the choice kernel $c(\neg a)$ for all other actions slowly decrease. (We show the precise mechanism of action-history module in the next section and in Fig. S6). When looking at the averages over participants (bottom), choice kernel c might differ between actions: $c("D")$ and $c("J")$ are potentially larger than $c("F")$ and $c("K")$. This would imply that—all else being equal—participants preferred some keys ("D" and "J") over others ("F" and "K"). F) Hidden state $s^{(r)}$. $s^{(r)}$ has 32 dimensions (hidden units). We performed a principal component analysis

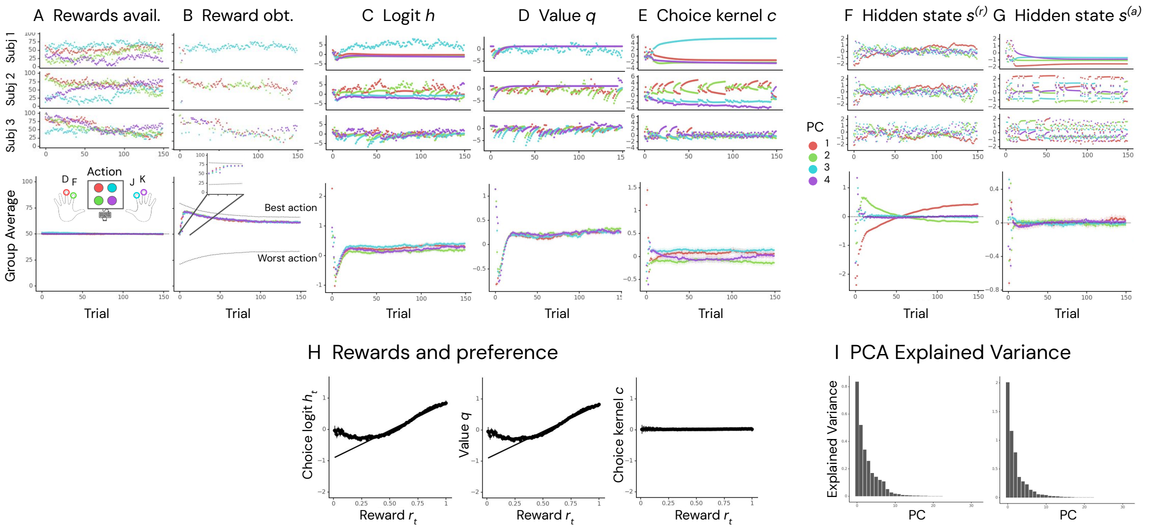

Figure 5: Latent Dynamics of Memory-ANN. A) Rewards available. The top part is replicated from main text Fig. 1E. The inset is a legend and applies to figure panels A-E. It shows which keys and which fingers were used to select which bandits on the screen (bandit stimuli were randomized between participants). The bottom part shows the mean reward schedule over all participants. Average rewards (colored lines) were the same for all bandits and trials, showing that reward schedules were not biased towards specific actions or trials. B) Reward obtained. The top part is replicated from main text Fig. 1E. The bottom part shows the average over participants. Participants started the task by winning the expected 50 points, but only took 5 or 6 trials (inset) to winning about 73 points (close to the theoretical—and unachievable—optimum). The black dotted lines show the average best and worst bandit, respectively, and indicate that through the process of Gaussian diffusion, rewards slightly regressed toward the mean over the course of a task. Even though the number of points won by participants decreased after the first dozen or so trials, the distance to the theoretical optimum remained roughly the same, suggesting that they were performing well throughout. C) Choice logits h. The top part of the figure shows the choice logits $h(a)$ for each action (color) for three example participants, derived from the winning model Memory-ANN. The model was fitted to each participant in a "closed-loop" fashion (see "Model Analysis" above) to obtain the individualized time courses. Logits h are the immediate precursors of action choice, separated only by a softmax transform. The time courses show a close correspondence to participants' observed actions (in panel B), and reveal potential individual differences. The bottom part of the figure shows averages over participants. Fluctuations over trials cannot be interpreted because the softmax is insensitive to differences in the mean and is calculated separately for each trial.

(PCA) and only show the first four principal components (PCs) here (part I of this figure shows each component's explained variance). PC1, "reward sensitivity" (red), was presented in the main text (Fig. 3F). Individual participants (top) show that reward sensitivity changes slowly over trials, with a tendency to increase when large rewards keep being obtained. Averages over participants (bottom) show systematic changes over trials, including a surge in reward sensitivity (PC1). G) Hidden state $s^{(a)}$. The same analyses, repeated for the hidden state of the action-history module. Top: A participant who persevered more (top) is predicted to experience more sustained states than a participant who repeatedly switched between actions (bottom). H) Rewards and choice logits. The logits $h_t(a)$ leading to an action a was significantly correlated with the reward r_t obtained for that action choice (left panel). This correlation was driven by the correlation between Q and reward (middle). c did not show a strong relationship with reward (right). I) PCA Explained Variance. Left: Explained variance by each principal component after performing PCA on the latent state $s^{(r)}$ of reward module. Right: Same for $s^{(a)}$. About 8-10 PCs explained most of the variance in both cases.

Action Processing Channel

Memory-ANN has two parts, reward module and action-history module. The Reward ANN maps rewards onto values Q, and was discussed in detail in the main text. The Action-history ANN maps actions onto choice kernel c, and will briefly be discussed now. The main difference between both is that reward module only updates the values $Q(a)$ of the chosen action a, whereas action-history module updates the entire vector of all four actions c on each trial. We analyzed action-history module in the same way as reward module, by extracting the fitted network parameters from the winning Memory-ANN, and probing a fresh instantiation of action-history module, based on these parameters, on the full range of inputs (see "Model Inspection"). The Action-history ANN takes two inputs: its own previous hidden state $s_{t-1}^{(a)}$ and

the previous action a_{t-1}. We first tested the response of the network when fixing $s^{(a)}_{t-1}$ to its average value, to assess the network's response in its most common setting, and only varied the second input, a. a can take on four different values: participants chose between the keys "D" and "F," operated with their left hand, and "J" and "K," controlled with their right. We first probed the action-history module with the action "D" (red circle, Fig. S6A, right). The response was a choice kernel vector with four elements, one for each action. The highest value was assigned to the action "D," which favors repetition of the same action (perseveration). The second-highest value was given to "F," the other key controlled by the same, left hand. The third and the last value were given to the two keys controlled by the other, right hand, "J" and "K." Both of these values were negative, hence leading the network to avoid these actions. When we probed action-history module with the other actions ("F", "J," and "K;" Fig. S6A, right), the same pattern arose three more times: The highest value was always given to same key (perseveration). The second value was always given to the other key controlled by the same hand. And the smallest values, which were always negative, were consistently given to the two keys operated by the other hand (Fig. S6A, right). This suggests that action-history module implements a consistent, abstract rule in the calculation of the choice kernel. This rule is largely independent of the particular action, and instead relies on the relationship between actions ("same action," "same hand," and "other hand").

We also tested action-history module after perturbing the hidden state $s^{(a)}_{t-1}$, to assess how this rule might change when the network operates in different settings. We perturbed the state by moving it along its first principal component, like we had done for reward module (see Fig. 3F). For action-history module, because of its four-dimensional output, we chose two points at which we probed the network: 1.5 standard deviations to each side of the mean activation $s^{(a)}_{t-1}$, along the axis of PC1. We found that the pattern of the choice kernel changed—but without affecting the rule: The ordering of actions remained intact; only their relative weights changed.

When we moved the hidden state in one direction, which we will call “right” for convenience, weights in the right hand became more extreme: The values for J after J and for K after K, which were already the largest weights in the average state, increased even more. The values for D or F after J or K, on the other hand, which already were the smallest weights in the average state, decreased even more (data not shown). The opposite pattern emerged when we perturbed the hidden state in the opposite, “left” direction: The values for the left hand became more extreme, with further surges in the values for choosing D after D and F after F, and further decreases in the values, already negative, of choosing K or J after D or F. In the “left” state, the values following left actions (D and F) are more extreme, repeating left actions more, and avoiding right actions more). In the “right” state, right actions are repeated more, and left actions are avoided more.

Taken together, action-history module seems to implement a hierarchical rule in the choice kernel, which favors repetition of the same action to switching actions within the same hand, and which favors staying within the same hand to switching to the other hand. This pattern is reminiscent of a hierarchical structure in which keys are nested within hands. This might reflect a bias in participants to strategically explore local actions rather than venture to far in action space. Future research will be needed to determine the cause of this pattern, and connect it to the wealth of existing research on hierarchical cognition and action sequence learning. The hidden state of the system $s_{t-1}^{(a)}$ hence does not affect the overarching rule, but how strong this rule is for a given hand.

We lastly asked how the outputs of both channels are combined. We initially fit weighting parameters to answer this question, which captured the relative weight of each channel ($h = \omega c + (1 - \omega) q$ or $h = \omega^{(a)} c + \omega^{(r)} q$). However, models that included weighting parameters did not converge unless we introduced further modifications (e.g., constraining outputs within a range). In the end, we chose the simplest implementation which did not constrain outputs

and did not include weighting parameters. Because outputs were unconstrained, the values they took on on each trial determined the relative weight of both channels. If c is larger than Q, for example, the sum h will be more heavily biased toward c. We quantified this intuition in the following way: Because the softmax is insensitive to differences in the mean (the result of $softmax([0, 1])$ is the same as the result of $softmax([10, 11])$), we first subtracted the mean values on each trial, separately for c and Q. After this step, both c and Q were centered on 0. We then took the absolute values of c and Q to assess the relative weight that each will have on the sum h. Finally, we averaged the weights over participants and visualized them over trials (Fig. S6B). We observed that the relative weights seemed to change over the course of the task: within the first dozen or so trials, the weight of the action channel increases rapidly, while the weight of the reward channel drops. We are planning to investigate this pattern in more detail in future work.

Effect of Training Data Size and Other Hyperparameters

Cognitive modeling studies typically use experimental datasets that contain between 20-50 participants. These samples sizes are chosen partly in continuity with existing studies in cognitive science, psychology, and neuroscience. Another factor are resource constraints, which generally make larger datasets more difficult to collect, and also to analyze. However, access to larger datasets could be a step change in understanding cognitive mechanisms. Smaller datasets have a lower noise ceiling because less frequent patterns might only occur once or a few times, and hence could be mistaken for random noise. Larger datasets can better identify systematic patterns from random noise because even infrequent events will occur at sufficient numbers to make them detectable. The noise ceiling is especially critical when studying complex systems: their processes are especially rich, nuanced, and varied, and hence especially difficult to detect in small datasets. Therefore, larger dataset might be paramount to understanding the details of

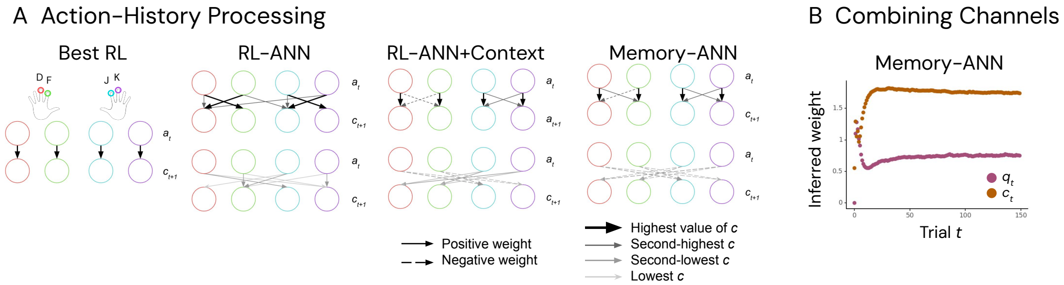

Figure 6: A) Action History Processing. The two sub-panels show action-history processing for the Best RL and the Memory-ANN model. The color legend is shown with Best RL: Each circle refers to one action available to participants (red: D, green: F, blue: J, and purple: K). Circles are arranged in a row, mirroring their positions on a standard keyboard. The top row of circles in each panel shows the input to the network (the previous choice a_t). The bottom row represents the network's output, the choice kernel for the next trial, c_{t+1}. Arrows indicate the relative order of the outputs. The thick black arrow points to the action that received the highest value; the thin black arrow points to the second-highest; and so on. Best RL (left) implements a simple perseveration rule, where the previous action receives a small additive bonus (see "Q-Learning Model Architectures"). Memory-ANN implements a more complex mapping, in which the value is highest for the same action, second-highest for the other action controlled by the same hand, and lowest for the actions controlled by the other hand. B) Combining Channels. To obtain the "inferred weight" of each channel, we first mean-centered each channel, separately for each trial t, and then took the absolute value: $|Q_t(a_i) - mean(Q_t)|$ and $|c_t(a_i) - mean(c_t)|$. Colored circles show the averages over participants for each channel. Error bars show the standard deviation.

cognition, arguably a rather complex process. This chain of reasoning is supported by the recent observation that a continuous increase in training data size, among other factors, has led to an apparent step change in model performance in the case of large-language models. Abilities that were previously thought impossible to learn from data, such as the mastery of grammar and fluency in a variety of subject matters, emerged once enough data was available.

We assessed what impact the size of the training dataset had on model performance in our study, for two reasons: 1) We were looking for evidence that larger datasets can indeed increase the noise ceiling, and allow us to capture more details about the cognitive mechanism. 2) We wanted to assess whether classic cognitive models and neural networks responded differently to increases in dataset size. We assumed that neural-network models would benefit more from additional data because they are more flexible, and can learn new patterns in a bottom-up way. To answer these questions, we retrained our five main models on smaller partitions of our original training dataset. We created five partitions, of sizes 32; 100; 300; 1,000; and 3,300 tasks. For comparability, we trained all models using the same hyperparameters (number of training steps: 500,000; optimizer learning rate: 0.0001; number of hidden units: 16; batch size: 32; L2 weight decay: 0.00001).

We found positive answers to both questions. 1) Training data size improved the loss of our most general model, Vanilla RNN (from 100.8 [$n = 32$] to 67.8 [$n = 3,300$], $t(412) = 31.5$, $p = 1.2e - 111$), and also of our winning model, Memory-ANN (from 81.7 [$n = 32$] to 63.9 [$n = 3,300$], $t(412) = 17.9, p = 1.4e - 53$) (Fig. S7A). This shows that flexible models indeed capture additional variance in human behavior just by being trained on more of it. Importantly, this improvement is not a symptom of overfitting because the improvement is not only evident in the training data (not shown), but generalizes to unseen tasks in the test data. 2) Indeed, the classic model Best RL did not improve much from larger datasets (loss of 84.8 [$n = 32$], compared to 82.3 [$n = 3,300$], $t(412) = 9.6$, $p = 7.2e - 20$). Hence, a third reason for small

sample sizes might be that classic cognitive models do not necessarily benefit from more data.

It is notable that Memory-ANN overfitted less than Vanilla RNN for small dataset sizes, and still outperformed it on larger datasets. This shows that it really captured the right bias in the data.

It is well known that neural networks typically require large amounts of data: if the training dataset is too small, networks "overfit" and fail to generalize to the testing data, which leads to an increased generalization loss. But not only the size of the dataset matters. Networks also overfit more when they are trained for longer, which gives them more opportunity to memorize the training data, and when they have more free parameters, which makes them more flexible and better able to mimic idiosyncrasies in the training data rather than learning a generalizable pattern. (In addition, exceptions exist to each of these rules of thumb, and other hyperparameters can also impact the fit.)

A model is tuned well to a dataset when all hyperparameters are chosen such that the network can absorb as much systematic variance as possible and as little noise as possible. Problems arise when the model is too flexible for a dataset (e.g., too many hidden units in the model; too little training data), or contrarily, when the model is too constrained (e.g., too few hidden units; large and complex dataset). Whereas the first problem leads to overfitting, the second would be a waste of valuable data resources. We wanted to make sure that we had tuned our models optimally for our dataset, such that we can hope to distill interesting cognitive mechanisms. To this end, we systematically assessed how training time (number of training batches) and model complexity (number of hidden units) affected model fits.

In both cases, the right choice of hyperparameters made a difference for model fit (Fig. S7B and C). In sum, we have created neural network models that make optimal use of our dataset. More flexible models were unable to distill more information from the data, showing that we have chosen the right model complexity. Even larger datasets are not expected to improve the

fit of our models, given the observed plateau in losses for increasing data sizes. While a larger dataset in conjunction with more flexible models might improve performance, we do not expect large improvements, based on the patterns we have observed.

Additional PCA Results

When we presented reward module in the main text, we analyzed the high-dimensional hidden state $s^{(r)}$ using PCA. Fig. 3F in the main text shows the results for the largest PC, which we called reward sensitivity. Fig. S8 shows the results for four more PCs. We already identified PC1, PC2, and PC5 as long-term components: These PCs retain reward information across the entire task (Fig. 5B). On a trial-by-trial level, these PCs have a big effect on the value Q, as seen in the large differences between different colors (top row, Fig. S8). This shows that PC1, PC2, and PC5 have a direct and large impact on choices. The short-term components, PC3 and PC4, on the other hand, barely impact values, as seen in the overlapping lines of all colors (top row, Fig. S8), revealing that these PCs barely impact choices. Instead, the main role of short-term components might be to modify the hidden state $s^{(r)}$. This is tentatively evident in the stronger effect (steeper curve) of PC4 (bottom row, Fig. S8). However, more detailed analyses would be required to systematically assess the effect on other PCs as well, and potentially confirm this suspicion.

Additional Model Fits

We ran a large number of models for this study and only show a small fraction in the main text. Suppl. Tables 2-8 show additional models. We include the fits of a subset of the Q-learning models we created to identify the Best RL model (Table S2). We also assessed the individual role of each processing channel (reward module and action-history module). We test how well each can explain human behavior on its own, reporting the fits of model variants that only

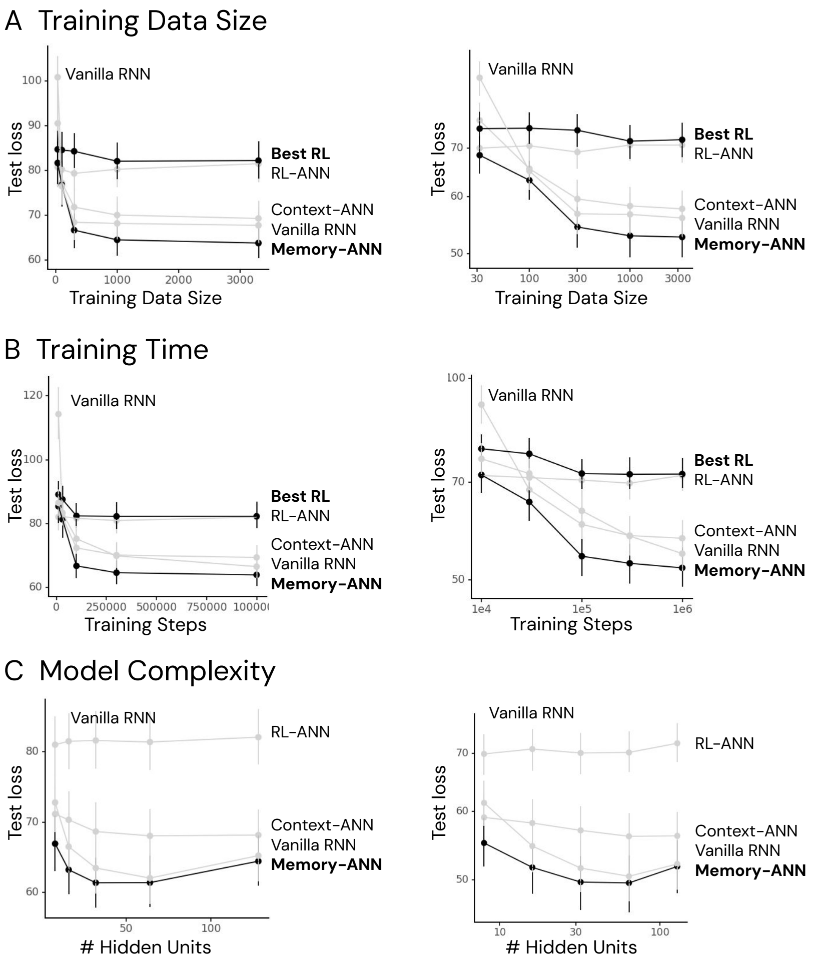

Figure 7: Effects of Training Data Size and Other Hyperparameters. A) Training Data Size. The left plot shows the cross-entropy loss of each model for differently-sized partitions of the training data. The right plot shows the same data on a log-log scale. The classic cognitive model Best RL and the winning model Memory-ANN are highlighted in black. Dots show the means and error bars show the standard deviations over tasks in each partition. B) Training Time. The same analysis as in part A, but assessing the effect of the number of training steps on model fit. C) Model Complexity. Assessing the effect of the number of hidden units in each model. This only applies to neural-network models, hence Best RL is excluded in this plot. Note that Vanilla RNN has one hidden layer, whereas RL-ANN, Context-ANN, and Memory-ANN have two hidden layers. We count the number of hidden units per layer. Hence, the hybrid models effectively have twice the number of hidden units, compared to Vanilla RNN. D) All other PCs (continuation of Fig. 3J-K). Most PCs showed minimal sensitivity to the past history. E) PC Summary. Short-term and long-term PCs had unique profiles in terms of the mean and maximum coefficients (from Fig. 3J-K). Long-term PCs (blue) showed large mean coefficients (averaged over trials) and large maximum coefficients (across trials); short-term PCs (red) showed high maximum coefficients, but low mean coefficients; most PCs (gray) had small maximum and mean coefficients.

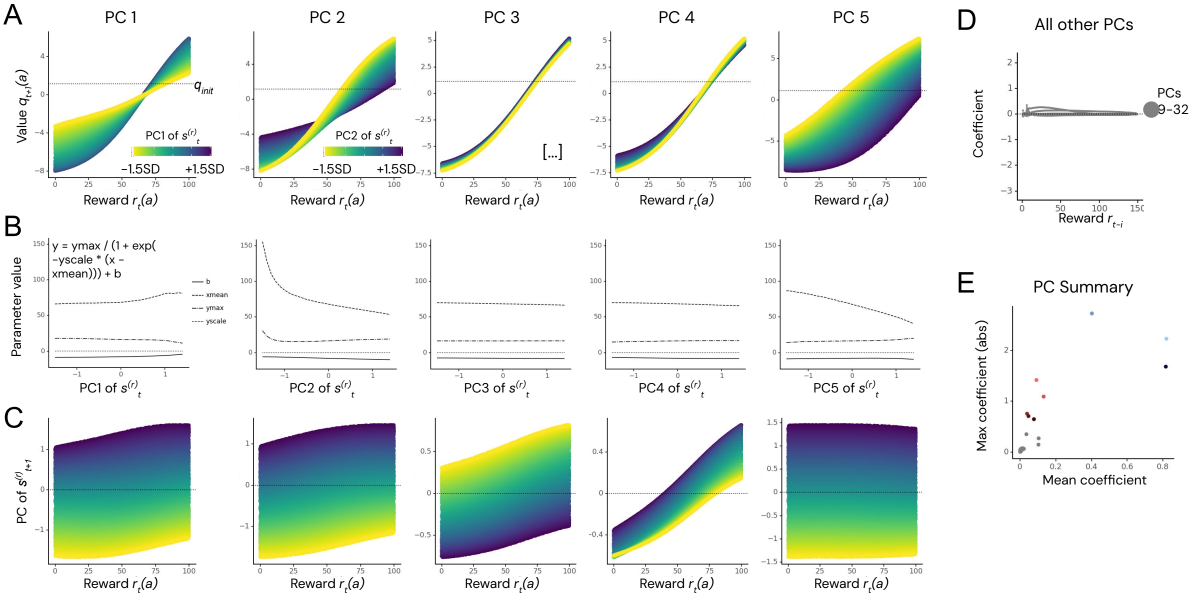

Figure 8: Effect of the hidden state on reward processing. Each column shows one PC of the hidden state $s^{(r)}$. A) Future values $Q_{t+1}(a)$ (y-axis) depend on past reward $r_t(a)$ (x-axis). This mapping is modulated by the hidden state $s_t(a)$. We visualize this modulation by showing different PCs of the hidden state (color). The PCs differ in how much they affect the mapping: PC1, PC2, and PC5 show qualitatively large effects, while PC3 and PC4 show qualitatively small effects. B) Parameters of the sigmoid functions. We fitted the parameters of a sigmoid function (see inset in the leftmost column) to the data shown in panel A. The fits were excellent in all cases (data not shown). This figure shows the fitted parameters, for different states. C) The model updated hidden states based on rewards r_t. Each column shows one PC.

contain one or the other channel (Table S3). We then focus on context representation, using either outputs or hidden states for conditioning. We built models that vary the representation independently for each channel, and such that use both context representations jointly (Table S4). We also assessed the importance of the forgetting parameter κ in the neural network models (Table S5). We then assessed potential interactions between reward processing and action-history processing, constructing models that have a single processing channel that receives both the reward r_{t-1} and the action a_{t-1} of the previous time step, similar to Vanilla RNN (Table S8).

Finally, we assessed the performance of other generic state-of-the-art sequence models (LSTM and transformer). We initially compared our models to simple RNNs rather than more advanced architectures in order to achieve the best experimental control for model comparison. Because we know exactly which elements differ between each pair of models, for example between Memory-ANN and Vanilla RNN, we can interpret the results of our model comparison as providing evidence for or against each of these differences. If we were to compare two architectures that differ in several ways, e.g., comparing Memory-ANN to a transformer architecture, we would lose this experimental control. In this sense, more advanced models will not provide the optimal comparison for the purposes of tight experimental control and interpretability of the findings.

However, more advanced architectures can still establish a useful upper bound on the predictive performance of our models for the current dataset. We hence fitted two new architectures to the current dataset, an LSTM and a transformer. We used similar loss functions and methods for hyperparameter tuning as before, training each model on the training split of our dataset and identifying the best architecture based on prediction performance on the validation split. The best LSTM had the following hyperparameters: Optimizer learning rate: 0.0001 Number of hidden units: 32 Batch size: 128 Weight decay: 0.0001 This LSTM achieved a prediction accuracy of 68.6% on the testing data, compared to 68.3% for our winning Memory-ANN (paired t-test:

t(412)=4.5, $p < 0.0001$) and 68.1% of the Vanilla RNN (paired t-test: t(412)=8.2, $p < 0.0001$; see Figure S9). This implies that it is possible to further improve upon the cognitive model we have proposed in terms of predicting the behavior of held-out participants. Nevertheless, the expected gains are relatively small compared to the improvements we have seen when moving from classic models to ANN-based models (Best RL achieved 60.6%; paired t-test: t(412)=29.4, $p < 0.0001$).

The reason that the LSTM model was able to improve upon the performance seen in the RNN-based models likely lies in the fact that it is more data efficient and better able to capture long-term dependencies in the data. Hence, future studies will need to determine how much of the improvement reflects an improvement in mirroring participants' cognitive processes, versus efficiency gains in fitting the model to a dataset of limited size.

We also fitted a transformer architecture to the current dataset. We used a different codebase to set up the training procedure, which means that the loss function and hyperparameter sweep were not exactly identical, though they were comparable.

The transformer achieved a prediction accuracy of 69.7%, compared to 68.3% for Memory-ANN, 68.1% of the Vanilla RNN (paired t-test: t(412)=8.2, $p < 0.0001$; see Figure S9), and 68.6% for the LSTM. Together, these results confirm that more sophisticated sequence models can outperform simpler RNNs, but not by much, compared to the improvement gains we obtained by moving from any of the simplest models (like Best RL and RL-ANN) to models with more flexible memory usage (like Memory-ANN, Vanilla RNN, LSTM, or Transformer). The relatively similar performance of all these models confirms that our models are approaching the asymptote of explainable variance in human behavior in our dataset.

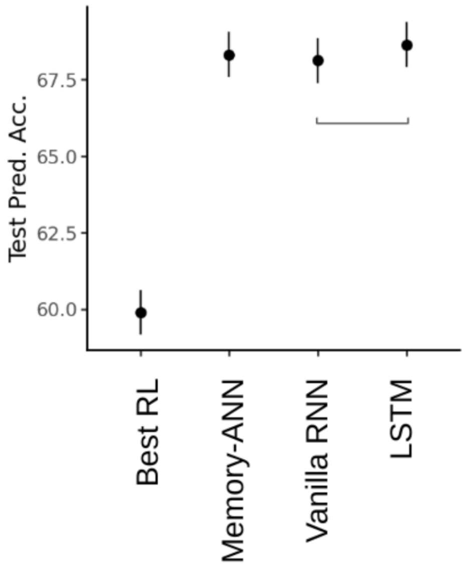

Figure 9: Model fits of generic state-of-the-art sequence models.

Table 2: Fits of a subset of classic Q-learning models. The Test Loss is in units of average trialwise log-likelihood, as described in the text. Prediction Accuracy (Pred. Acc.) refers to the conversion of this number into a percentage, as described in the text.

**Model Name**	**Parameters**	**Test Loss**
	α	181.9
Basic RL	α, β	98.7
	α, β, b	96.9
	α, β, b, κ	84.3
	$\alpha, \beta, b, \kappa, Q_{init}$	84.4
Best RL	$\alpha, \beta, b, \kappa, Q_{init}, p$	82.2
Variable α	$\alpha_{init}, \beta, b, \kappa, Q_{init}, p, w$	80.8

Table 3: Fits (test loss) of models that consist of only one processing channel (reward processing only or action-history processing only). We show both the "classic variant" (component of Best RL that processes action history or rewards) and the corresponding neural-network channel (reward module or action-history module of each neural-network model presented in the main text).

**Model Description**	**Test Loss**
Reward Processing Only	
Classic variant ($\alpha, \beta, b, \kappa, Q_{init}$)	84.4
Reward ANN (as part of RL-ANN)	83.3
Reward ANN (as part of Context-ANN)	80.1
Reward ANN (as part of Memory-ANN)	73.5
Action-History Processing Only	
Classic variant (p)	141.2
Action-history ANN (as part of RL-ANN)	108.7
Action-history ANN (as part of Context-ANN)	80.5
Action-history ANN (as part of Memory-ANN)	74.7

Table 4: The reward module and the action-history module of the same model can have different context representations (conditioning on outputs versus hidden states). The column "Reward ANN" ["action-history module"] specifies the context representation of the reward module [action-history module], by reference to the model in the main text with the same structure. "RL-ANN" means that no additional information is passed; "Context-ANN" means that the previous output is passed; and "Memory-ANN" means that the hidden state is passed. "Both" indicates that the model receives both its previous output and its previous hidden state.

**Reward Module**	**Action-History ANN**	**Test Loss**
RL-ANN	Context-ANN	69.6
RL-ANN	Memory-ANN	66.2
Context-ANN	RL-ANN	77.7
Context-ANN	Memory-ANN	64.9
Memory-ANN	RL-ANN	71.8
Memory-ANN	Context-ANN	64.3
Both	Both	61.6

Table 5: Fit of neural-network models trained without forgetting (no hyperparameter tuning).

**Model**	**Test Loss**
RL-ANN ($\kappa = 0$)	81.1
Context-ANN ($\kappa = 0$)	68.6
Memory-ANN ($\kappa = 0$)	62.9

Table 6: Model losses and prediction accuracy of models presented in the main paper.

**Model**	**Test Loss**	**Pred. Acc.**
Best RL	82.3	60.6%
RL-ANN	81.5	60.8%
Context-ANN	67.9	65.4%
Memory-ANN	61.3	68.3%

Table 7: Testset losses of generic sequence models.

**Model**	**Test Loss**
Vanilla RNN	61.7
LSTM	60.4
Transformer	58.4

Table 8: Fits of models with a single processing channel (no hyperparameter tuning).

**Recurring Variable**	**Model Description**	**Test Loss**
Hidden state s_{t-1}	Vanilla RNN	61.4
Output h_{t-1}	(Like Context-ANN)	69.4
Nothing	(Like RL-ANN)	93.0

Table 9: Fits (test loss) of model variants with simplified action-processing module. In these variants, the action module only has one output node, like the reward-processing module, which updates the choice kernel $c(a)$ of the chosen action a. The values $c(\neg a)$ of all other actions decay towards c_{init} (a fitted parameter), at a rate of κ_c (a fitted parameter).

**Model Description**	**Test Loss**
Otherwise like RL-ANN	78.9
Otherwise like Context-ANN	73.5
Otherwise like Memory-ANN	67.6

Additional Qualitative Model Analyses

The main paper focuses on a subset of models and behavioral patterns. Fig. S10 and S11 provide a more comprehensive summary.

Causal Manipulation on Latent Model States

Our first set of analyses is targeted at better understanding the timescales of learning and reveals how the latent state supports this. We performed an in-silicio experiment to test what happens if Memory-ANN lacks the recurrent state. 1) We artificially clamped the reward module's state to a fixed value, which effectively reduces the module to a lookup table that maps each reward magnitude onto one specific Q-value. This "memory-less" reward module does not have the ability to adjust processing to the current task context—its behavior is stable over time. 2) We simulated the behavior of a Memory-ANN with such a fixed reward module, clamping its memory state to the same value for the entirety of the task. The model's task behavior was surprisingly intact, but lacked crucial features of human behavior. As predicted by the reviewer, the lesioned model's behavior became qualitatively more similar to Best RL.

Having shown that without a latent state, Memory-ANN loses its ability for context-sensitive reward processing, we next dissect what this ability entails and how it is implemented. 3) We first assessed the effects of time on latent model states in the absence of any causal manipulations, using in-silico simulations and recordings. We found that the same numeric rewards resulted in lower Q-values on early compared to late trials. Because the reference value Qinit stays the same throughout the task, the role of this mechanism might be to promote the selection of unchosen actions early on ("exploration") and shift the mechanism to focus on high-valued choices later ("exploitation"). 4) We then plotted raw model states depending on actual reward histories, creating various reward sequences and recording the resulting module states. We find that even a single reward usually has long-term, non-linear, and oftentimes non-monotonic ef-

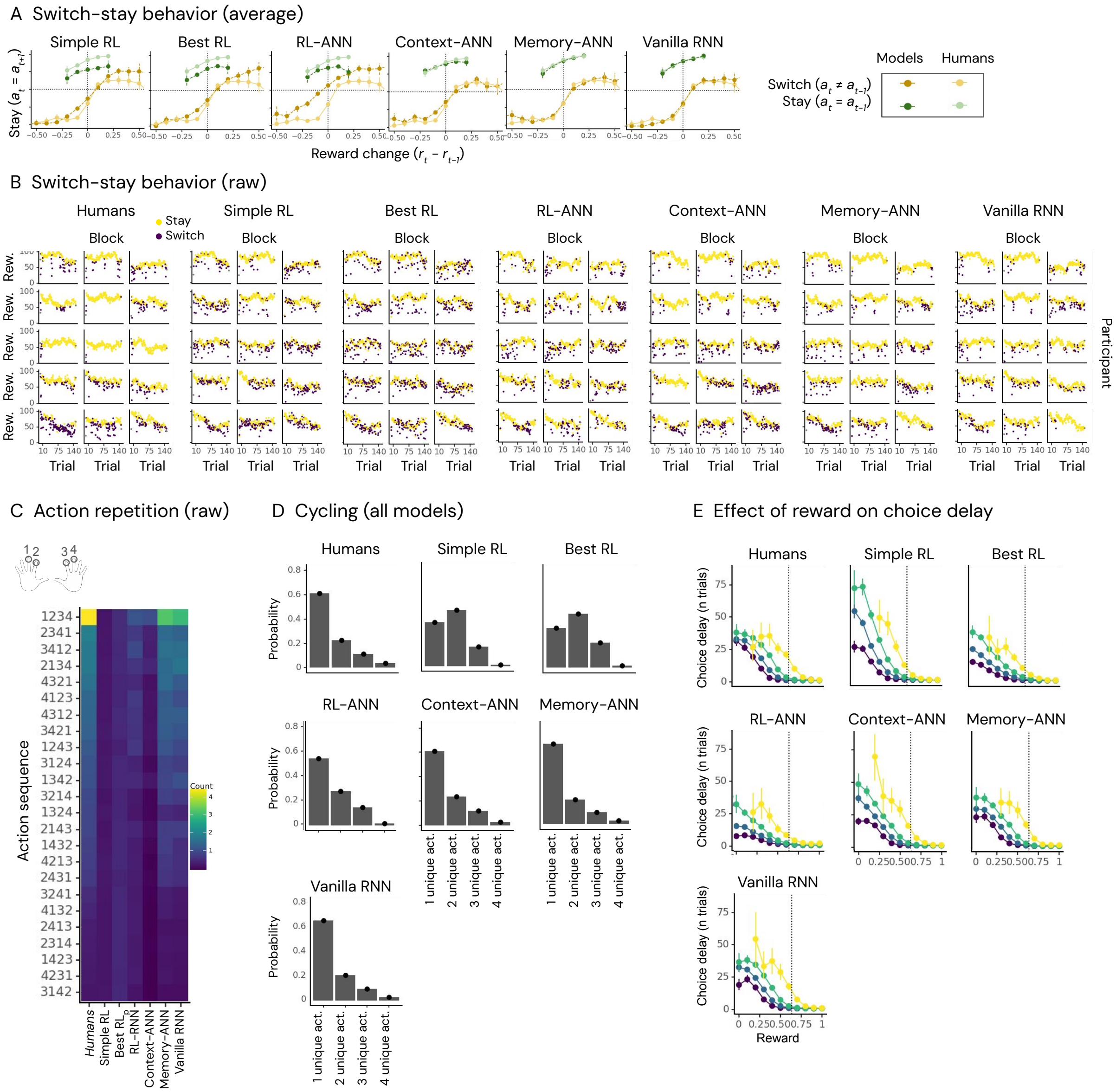

Figure 10: Additional behavioral analyses. A) Switch-stay behavior (average). Humans (lighter colors) are shown in each subpanel for reference. B) Switch-stay behavior (raw). The subpanel for each model (and humans) shows five example participants (rows) who each perform three blocks of the task (columns). Each trial's obtained reward magnitude ("rew.") is shown on the y-axis, and the color denotes where a trial was a stay (same action as on the previous trial) or a switch (different action than on the previous trial). C) Action repetition (raw). We counted the number of all action sequences of length four in which each of the four actions was chosen exactly once. The y-axis shows each possible sequence, sorted by their frequency (color) in human behavior. Different models are shown in columns. While Simple RL and Best RL are not able to capture any sequential patterns, RL-ANN and Context-ANN show the ability to capture sequences of length 2. Only Memory-ANN and Vanilla RNN qualitatively capture the full spectrum of repetition preferences. D) Cycling (extended). We counted, for each sequence of four actions, how many unique actions were performed. Instances where only 1 action was performed are called "multiple-repeats" in the main text. Instances where all 4 actions were performed are called "cyclic responses". This figure additionally shows the frequency of sequences with 2 and 3 unique actions. E) Effect of reward on choice delay. Choice delay was measured as the number of trials until the same action was chosen again, after receiving the reward shown on the x-axis.

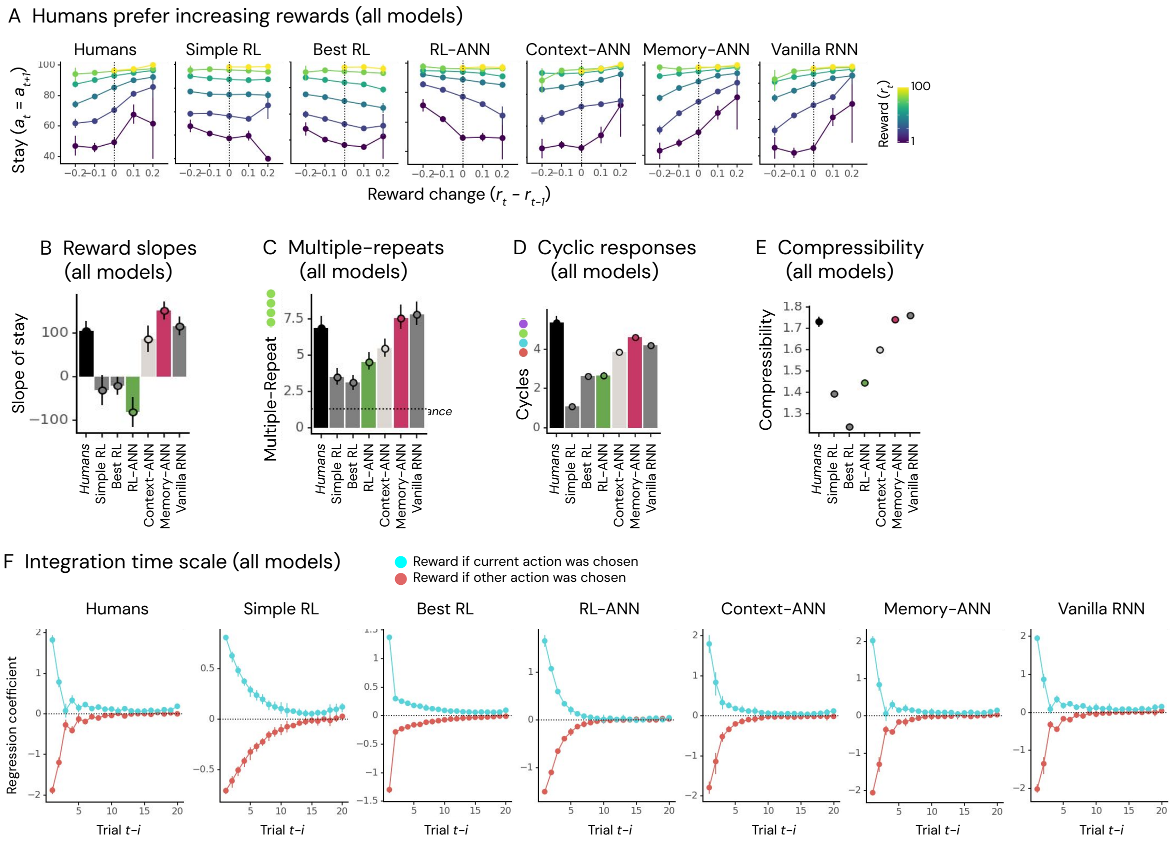

Figure 11: Behavioral analyses presented in main text Fig. 4, shown for all main models. A) Humans prefer increasing rewards (all models). B) Reward slopes (all models). C) Multiple-repeats (all models). D) Cyclic responses (all models). E) Compressibility (all models). F) Integration time scale (all models).

fects on the state, especially if the reward is sufficiently different from previous rewards. We also assessed long-term reward dynamics by creating a series of continuously changing reward sequences. We found that gradual changes in rewards also affected latent states, with continuous variations between sequences based on their direction (e.g., increasing / decreasing) and rate (fast / slow). 5) Finally, we used targeted causal manipulations to assess the long-term effects of different latent states on the subsequent calculation of Q-values. We obtained states of interest by feeding the reward module different reward histories and injected these states into fresh networks, examining the resulting behavior. Whereas network states associated with a history of low rewards led to an inflation of subsequent Q-values, network states associated with a history of high rewards led to a depression of subsequent Q-values. These effects lasted for dozens of trials and might support continued task engagement in the face of fluctuating rewards.

We lastly zoomed in on the role of different state PCs. 6) We individually disrupted and biased each PC dimension to understand its causal role. PCs had qualitatively different effects, for example increasing / decreasing Q-values or equating / exaggerating differences between rewards, suggesting that the reward state can affect reward processing in various ways and at multiple timescales. We describe the details of each of these analyses in the following.

1) If Memory-ANN's latent state was fixed over time, each different reward magnitude would be mapped onto one particular Q-value, independent of the previous history of rewards. To visualize this, we extracted Memory-ANN's reward module using the same method as in the main paper, set the state $s^{(r)}$ to the average value observed in humans, and probed the resulting module across the range of allowed rewards, for 150 trials. As expected, such a "memory-less" module reduces to a simple look-up table that maps each reward to a particular Q-value (Fig. S12A).

2) We then simulated behavior from a population of Memory-ANN agents with such a "clamped" reward module. Overall task performance was only slightly reduced (Fig. S12B1),

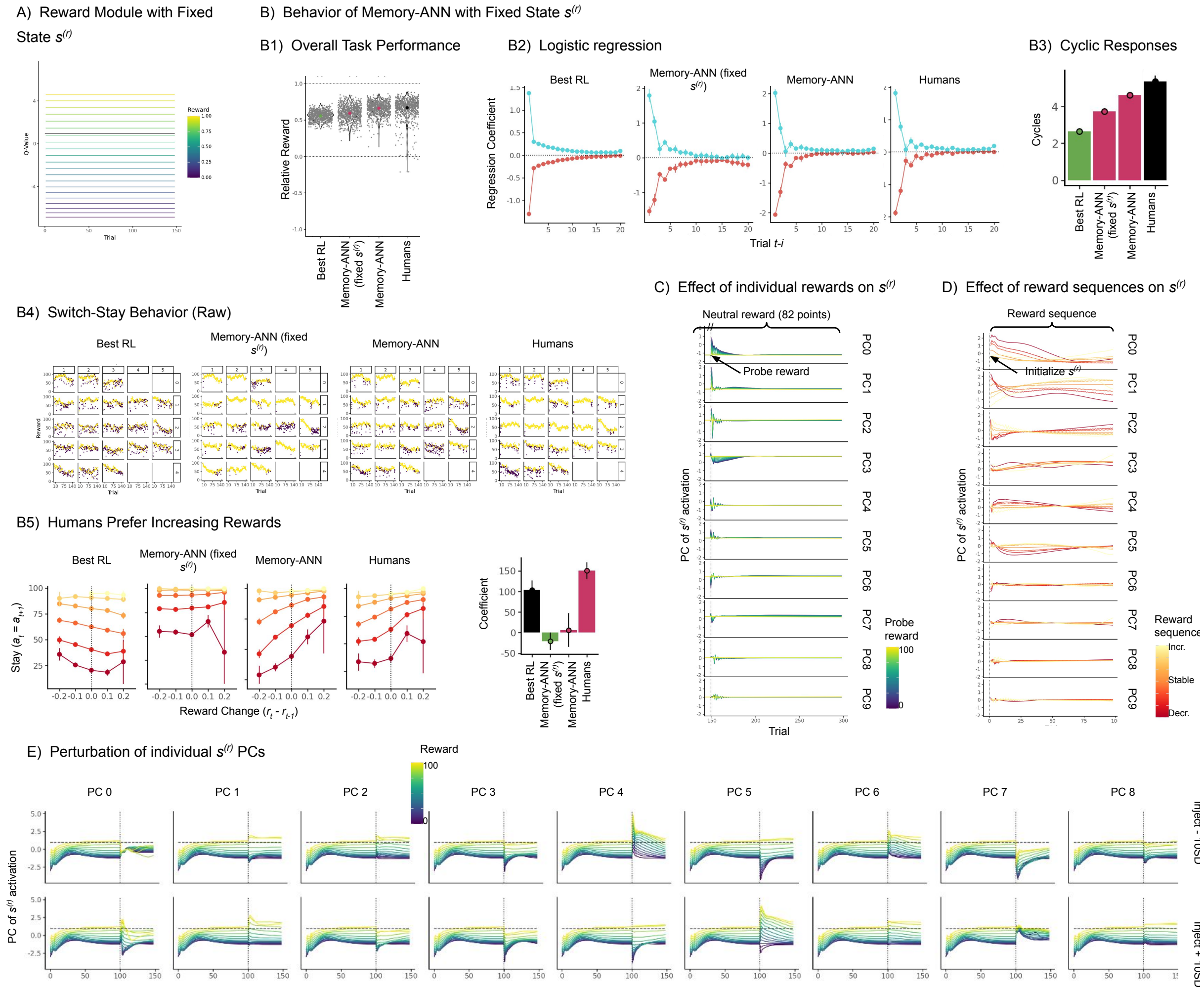

Figure 12: A) Reward module with fixed $s^{(r)}$. B) Behavior of Memory-ANN with fixed state $s^{(r)}$. B1) Overall task performance. B2) Logistic regression. B3) Cyclic responses. B4) Switch-stay behavior. B5) Humans prefer increasing rewards. C) Effects of individual rewards on $s^{(r)}$. D) Effect of reward sequences on $s^{(r)}$. E) Perturbation of individual $s^{(r)}$ PCs.

but specific patterns of behavior were qualitatively altered, often rendering it more similar to Best RL. This was evident in the qualitatively smoother effect of past rewards on subsequent choices (Fig. S12B2), reduced occurrence of cyclic response patterns (Fig. S12B3), more condensed switch-stay behavior (Fig. S12B4), and in the neutral slope of the effect of recent reward changes on behavior (Fig. S12B5). This shows that without the ability to adjust behavior to the reward context, Memory-ANN shows a series of qualitative behavioral changes that make it qualitatively more similar to Best RL, but qualitatively less similar to humans.

3) We next assessed the mechanism more closely by which $s^{(r)}$ modulates the calculation of Q-values, probing how the reward module responds to the same rewards at different time points during the task. To this aim, we isolated the reward module of a fully-trained Memory-ANN and probed it with various inputs while recording its outputs, using the same methods as in the main paper. To understand differences in processing over time, we probed the network with sequences of 150 identical rewards across the range of rewards, and identified changes in its output over time. We observed that rewards across the range of allowed magnitudes led to relatively low Q-values early-on in the task, but quickly increased within the first few dozen trials. For the next few dozen trials, Q-values fanned out relative to each other, such that smaller rewards led to increasingly smaller Q-values and larger rewards onto larger Q-values. This pattern stabilized about half-way through the task and remained comparatively stable until the final trial (see main text Fig. 3G). To understand the effect of these changes, it helps to take into account the reference value Q_{init}, which is stable for the entire task (dotted line). In order to select between choice options, Memory-ANN enters the Q-values of all four actions into a softmax to obtain choice logits, and eventually sampling probabilities. The Q-value of the most recently chosen action will correspond to one of the colored lines in Fig. 3G, based on the observed reward and trial. The Q-values of all other actions will either be Q_{init} (if the action has not yet been chosen) or at a value between Q_{init} and the Q-value corresponding to the previous outcome

when taking this action, which values closer to Q_{init} the longer the action has not been chosen for (if the action has been chosen before). Hence, when the reward module returns Q-values that are lower than Q_{init}, recently chosen actions face a disadvantage compared to actions that have not been chosen recently and whose Q-values had the chance to increase back towards Q_{init}. When the reward module returns Q-values that are higher than Q_{init}, on the other hand, recently chosen actions have an advantage over non-chosen ones. Hence, an overall increase in Q-values can facilitate a transition from more "exploratory" choice behavior (favoring recently unchosen actions) to more "exploitative" behavior (favoring high-reward actions), especially when only the highest rewards lead to Q-values that are larger than Q_{init}. This suggests that one role of $s^{(r)}$ is to capture changes in participants' processing of rewards over the extended time scale of a task, potentially shifting the balance from more exploratory choices to more exploitative ones. This analysis does not, however, differentiate between the number of trials spent on the task and the model's adjustment to specific reward magnitudes, which were confounded in the above analysis. We address this issue in point 5 below.

4a) We next tested how individual rewards impact the state $s^{(r)}$, as suggested by the reviewer. To this aim, we tested the effects of individual probe rewards that were outliers within sequences of otherwise identical rewards, on the state $s^{(r)}$. To do this, we fed the reward module 150 trials of the same reward (we arbitrarily chose 82 points, but confirmed that the results were consistent for other numbers) to stabilize the latent state. On trial 151, we subjected the module to a probe reward between 0 and 100 points, before going back to feeding it 82 points as before. We assessed the effect of this sudden change in reward magnitude on $s^{(r)}$, measuring its PCs. Note that relatively stable sequences with individual outlier rewards naturally occur in the current task because all rewards enter the reward module in the same way, independent of which action led to the reward. For example, if action A leads to an average of 82 points, but action B leads to 50 points, the reward module might experience a sudden change from 82 to

50 points if the participants consistently selects action A and then briefly checks in on action B. As expected, this manipulation produced a mix of long-term effects (e.g., PC0, PC1, PC3, PC5, PC7; Fig. S12C) and short-term effects on $s^{(r)}$ (e.g., PC2, PC6, PC8): The probe rewards affected subsequent states for one or two up to many dozens of trials, depending on the PC, and sometimes showed the largest effects many trials in the future (e.g., PC8). The strength of the effect of probe rewards scaled continuously with the distance between the neutral reward and the probe reward. This analysis further suggests that individual rewards impact the reward state $s^{(r)}$ at various timescales. (Note that the specific version of Memory-ANN presented here is different from the one presented in the main text in that it was fitted later and based on a different random seed, leading to slightly different model weights. Hence, individual PCs are not comparable between this figure and others).

4b) We next tested how long-term reward dynamics impacted the state $s^{(r)}$. To this aim, we subjected the reward module to increasing, stable, or decreasing reward sequences with different rates of change. The stable reward sequence provided 100 rewards of 50 points to the network. There were three levels of increasing reward sequences: fast (the first trial gave 1 point and the last 100, with 1-point increments on each trial); medium (the first trial gave 25 points and the last 75, with 1-point increments on every other trial); and fast (the first trial gave 33 points and the last 67, with 1-point increments on every third trial). Similarly, there were three levels of decreasing reward sequences: fast (100 to 1, 1 point decrease on each trial); medium (75 to 25, 1 point decrease on every other trial); and slow (67 to 33, 1 point decrease on every third trial). We initialized the networks to their average state in the human data to dissociate the effects of reward sequences from the long-term drifts identified in section 3). The analysis revealed a range of effects on s(r) (Fig. S12D). For example, PC1 seemed to show the largest response within the first 10-20 trials and then settled into a relatively stable state based on the rate of change, which stayed comparatively similar for the remainder of the experiment. PCs 4

and 5, on the other hand, show continued adjustment based on the reward magnitudes of each experiment. This suggests that the speed and direction of reward sequences might be encoded in state PCs.

5) We next assessed the causal role of $s^{(r)}$ for the calculation of Q-values, injecting different states into the reward module and recording the effects. Specifically, we first obtained a series of relevant states by subjecting the reward module to sequences of 150 identical rewards varying across the range of allowed rewards. We then created a fresh module, initialized it at each of these states, and then probed it for 150 trials on a "neutral" reward of 82 points (arbitrarily chosen; the results are robust to differences in this choice). We found that the reward module assigned larger Q-values to the neutral reward when it was initialized in a "poor" context (i.e., acclimatized to low rewards; purple lines; main text Fig. 3H) than when it was initialized in a "rich" context (acclimatized to high rewards; bluish-white lines; Fig. 3H). The effects of context initialization lasted for up to 50-100 trials, with potential differences based on the initial context. This analysis shows that one crucial role of $s^{(r)}$ is to adjust Q-value calculation to the established context of previous rewards. Similar processes have been proposed in the literature, and formalized, e.g., using value normalization frameworks (e.g., Bavard et al., 2018).

We now turn to our analyses assessing the roles of specific PCs. Please note that technical difficulties prohibited us from running the model with the originally trained weights. We hence fitted new models for these supplemental analyses, which showed similar overall behavior and reproduced all main results, but individual PCs were not the same. 6) We first assessed the causal roles of various PCs by performing perturbation experiments, i.e., by injecting artificial activation into one PC at a time. Like before, we extracted Memory-ANN's reward module and subjected it to sequences of 150 identical rewards across the range of possible rewards, allowing it to settle into a stable state $s^{(r)}$. On trial 151, we modified a single PC by modifying the state representation to set that PC to an extreme value (its average plus or minus ten standard

deviations) while leaving the values of all other PCs unaltered. We probed the module for another 150 trials on the same reward as before and observed the effects of these perturbations on the calculation of Q-values. We found that such perturbations to many PCs (e.g., PC0, PC3, PC4, PC5, PC7) caused long-term effects on the calculation of Q-values. For example, when we disrupted PC0 by artificially reducing its activation (a), all rewards led to similar, relatively low Q-values on the subsequent trial and only slowly recovered their differentiation, suggesting that PC0 plays a role in differentiating reward magnitudes (Fig. S12E). When we increased activations in PC0, on the other hand (b), the differentiation between rewards was exaggerated (but quickly went back to normal). When PC4 was injected with extreme activations, Q-values for all reward magnitudes either decreased (a) or increased (b) dramatically, while PC5 showed the inverse pattern (Fig. S12E). In sum, various PCs of $s^{(r)}$ showed short- and/or long-term effects on the subsequent calculation of Q-values.

Supplementary Discussion

In the era of machine learning, the main advantage of classic cognitive models is their interpretability: Models sketch the cognitive architecture of a thought process, detailing which pieces of information are processed by which modules, and in what order (think of classic boxes-and-arrows diagrams). Models also specify which operations or cognitive algorithms each module performs (for example, how a value is updated based on new information). Lastly, models make the waxing and waning of internal cognitive variables transparent, providing a direct glimpse at the underlying dynamics, an aspect that makes the method especially cherished in brain studies.

Like a classic cognitive model, Memory-ANN provides these three forms of insight. It draws the clear picture of a cognitive architecture that neatly separates reward processing from action-history processing (Ashby et al., 2007; K. J. Miller et al., 2019). Our method provides

new evidence for this separation that supports it in its most general form: independently of which algorithms are assumed to perform the processing, the best-fitting model shows that rewards and actions are processed separately. This dissociation is evocative of the classic distinction between model-free RL and other forms of learning, such as Hebbian or model-based learning: whereas model-free RL performs its updates based on reward information, Hebbian learning (K. J. Miller et al., 2019) and model building (Daw et al., 2006) use information other than reward in order to create a richer understanding of the environment. Partitioning the cognitive architecture by letting different modules see different aspects of the data, might hence point to a basic computational—and potentially biological—distinction. "Dual-process" models, which hold that human behavior is driven in concert by two separable processes, have a long tradition in the cognitive sciences (Dickinson, 2012; Dolan and Dayan, 2013).

Memory-ANN also offers a grasp of the cognitive algorithms that support human reward learning. The model reveals that algorithms operate at two levels of abstraction: The "surface" set of update rules processes the task observables (rewards and previous actions) and proposes which actions to select next (in the form of choice logits). These decision rules operate at the level of most existing models of cognition, but ours is a much simpler, direct mapping from rewards to Q-values. In its simplicity, this rule is reminiscent of basic perceptual decision making. No intermediate variables need to be calculated, not even prediction errors, the heart of classic delta-rule models. It is perhaps surprising that we can capture the full complexity of human reward learning in our task using this simplest of choice functions.

This works because the model contains a second, "deeper" layer of update rules, which operate entirely within the model's own latent representations. These update rules perform complex operations at multiple timescales, and construct rich latent representations that transform the continuous stream of observable task information into a buffet of potentially useful contextual knowledge. Identifying this second, crucial memory system complements a wealth of recent

findings in which humans show not just "surface-level" RL processes (Lee et al., 2012; Niv, 2009) but also evidence for a variety of memory systems operating on a variety of timescales, including working memory (Collins and Frank, 2012) and episodic memory (Bornstein and Norman, 2017; Duncan et al., 2018). One caveat with our methods is that these "deep" representations may capture not only the contents of memory, but also any individual differences in strategy between participants (Dezfouli et al., 2019), which our current approach is not designed to disentangle from differences in memory state over time.

Surface-level and deep processes come together in the form of contextualization: The simple choice rule can be flexibly modified based on the deep knowledge representing the current task context. The range of responses can be increased or reduced, and the system can be more or less sensitive to differences in reward magnitudes. In this way, one simple choice rule can reproduce complex human task behavior. Indeed, it is becoming increasingly evident how important context is for learning, both using cognitive modeling and RL, and classic methods. Our results underline how central contextual processing is, and contribute a precise sketch of its mechanism: the modulation of a simple choice rule based on rich, deep representations. This intertwining of learning systems, which operate at different levels of abstraction, echoes ideas from meta-learning, where new learning algorithms are discovered within the framework of an existing learning algorithm (Binz et al., 2023; Wang et al., 2018. Such intertwined learning systems also occur in hierarchical Reinforcement Learning, where learning within a single agent occurs in parallel at different levels of abstraction (Botvinick, 2012; Eckstein and Collins, 2020. The structure of the brain seems to support both a gradient of abstraction, and the intertwining of levels.

Most importantly, our findings make clear that deep representation learning and contextualization are at the core of human reward learning. These mechanisms are not an appendix to shallow mechanisms, but the two mechanisms that explain most additional variance in human

behavior. A similar insight has emerged independently in machine learning research on RL: rich representations, deep models, and complex algorithms are indispensable to create artificial systems that act adaptively in a world of real-world complexity. Hence, complexity might be indispensable in the explanation of human behavior, as it is in the creation of systems that act in a complex world. Even though we have only studied a single task here, it would be intriguing to see whether these patterns generalize to other tasks, including the vast literature on learning and beyond.

References

Ashby, F. G., Ennis, J. M., & Spiering, B. J. (2007). A neurobiological theory of automaticity in perceptual categorization. *Psychological Review*, *114*(3), 632–656.

Bavard, S., Lebreton, M., Khamassi, M., Coricelli, G., & Palminteri, S. (2018). Reference-point centering and range-adaptation enhance human reinforcement learning at the cost of irrational preferences [Publisher: Nature Publishing Group]. *Nature Communications*, *9*(1), 4503.

Binz, M., Dasgupta, I., Jagadish, A., Botvinick, M., Wang, J. X., & Schulz, E. (2023). Meta-Learned Models of Cognition.

Bornstein, A. M., & Norman, K. A. (2017). Reinstated episodic context guides sampling-based decisions for reward. *Nature Neuroscience*, *20*(7), 997–1003.

Botvinick, M. (2012). Hierarchical reinforcement learning and decision making. *Current Opinion in Neurobiology*, *22*(6), 956–962.

Collins, A. G. E., & Frank, M. J. (2012). How much of reinforcement learning is working memory, not reinforcement learning? A behavioral, computational, and neurogenetic analysis: Working memory in reinforcement learning. *European Journal of Neuroscience*, *35*(7), 1024–1035.

Daw, N. D., O'Doherty, J. P., Dayan, P., Seymour, B., & Dolan, R. J. (2006). Cortical substrates for exploratory decisions in humans [Number: 7095 Publisher: Nature Publishing Group]. *Nature*, *441*(7095), 876–879.

Dezfouli, A., Ashtiani, H., Ghattas, O., Nock, R., Dayan, P., & Ong, C. S. (2019). Disentangled behavioural representations. *Advances in Neural Information Processing Systems*, *32*.

Dickinson, A. (2012). Associative learning and animal cognition. *Philosophical Transactions of the Royal Society B: Biological Sciences*, *367*(1603), 2733–2742.

Dolan, R. J., & Dayan, P. (2013). Goals and Habits in the Brain. *Neuron*, *80*(2), 312–325.

Duncan, K., Doll, B. B., Daw, N. D., & Shohamy, D. (2018). More Than the Sum of Its Parts: A Role for the Hippocampus in Configural Reinforcement Learning. *Neuron*, *98*(3), 645–657.e6.

Eckstein, M. K., & Collins, A. G. E. (2020). Computational evidence for hierarchically structured reinforcement learning in humans. *Proceedings of the National Academy of Sciences*, *117*(47), 29381–29389.

Ji-An, L., Benna, M. K., & Mattar, M. G. (2024). Discovering Cognitive Strategies with Tiny Recurrent Neural Networks [Pages: 2023.04.12.536629 Section: New Results].

Katahira, K. (2024). Excessive flexibility? Recurrent neural networks can accommodate individual differences in reinforcement learning by capturing higher-order history dependencies.

Lampinen, A. K., Chan, S. C. Y., Singh, A. K., & Shanahan, M. (2024). The broader spectrum of in-context learning [arXiv:2412.03782 [cs]].

Lee, D., Seo, H., & Jung, M. W. (2012). Neural Basis of Reinforcement Learning and Decision Making. *Annual review of neuroscience*, *35*, 287–308.

Li, J., Schiller, D., Schoenbaum, G., Phelps, E. A., & Daw, N. D. (2011). Differential roles of human striatum and amygdala in associative learning. *Nature Neuroscience*, *14*(10), 1250–1252.

Miller, K., Eckstein, M., Botvinick, M., & Kurth-Nelson, Z. (2023). Cognitive Model Discovery via Disentangled RNNs. *Advances in Neural Information Processing Systems*, *36*, 61377–61394.

Miller, K. J., Shenhav, A., & Ludvig, E. A. (2019). Habits without Values. *Psychological review*, *126*(2), 292–311.

Niv, Y. (2009). Reinforcement learning in the brain. *Journal of Mathematical Psychology*, *53*(3), 139–154.

Pearce, J. M., & Hall, G. (1980). A model for Pavlovian learning: Variations in the effectiveness of conditioned but not of unconditioned stimuli. *Psychological Review*, *87*(6), 532–552.

Peterson, J. C., Bourgin, D. D., Agrawal, M., Reichman, D., & Griffiths, T. L. (2021). Using large-scale experiments and machine learning to discover theories of human decision-making. *Science*, *372*(6547), 1209–1214.

Song, M., Niv, Y., & Cai, M. (2021). Using Recurrent Neural Networks to Understand Human Reward Learning. *Proceedings of the Annual Meeting of the Cognitive Science Society*, *43*(43).

Wang, J. X., Kurth-Nelson, Z., Kumaran, D., Tirumala, D., Soyer, H., Leibo, J. Z., Hassabis, D., & Botvinick, M. (2018). Prefrontal cortex as a meta-reinforcement learning system. *Nature Neuroscience*, *21*(6), 860–868.
