# Supplementary material for: Hybrid neural–cognitive models reveal how memory shapes human reward learning
Source: Nat Hum Behav. 2026 Feb 5;10(5):972–87. doi: 10.1038/s41562-025-02324-0 (PMC13212159; doi:10.1038/s41562-025-02324-0)
Supplement: Supplementary file 2 — Reporting Summary [file 41562_2025_2324_MOESM2_ESM.pdf]

nature portfolio

Corresponding author(s): Maria Eckstein, Kevin Miller

Last updated by author(s): Oct 18, 2024

Reporting Summary

Nature Portfolio wishes to improve the reproducibility of the work that we publish. This form provides structure for consistency and transparency in reporting. For further information on Nature Portfolio policies, see our Editorial Policies and the Editorial Policy Checklist.

Statistics

For all statistical analyses, confirm that the following items are present in the figure legend, table legend, main text, or Methods section.

n/a	Confirmed	
☐	☒	The exact sample size (*n*) for each experimental group/condition, given as a discrete number and unit of measurement
☐	☒	A statement on whether measurements were taken from distinct samples or whether the same sample was measured repeatedly
☐	☒	The statistical test(s) used AND whether they are one- or two-sided<br>*Only common tests should be described solely by name; describe more complex techniques in the Methods section.*
☐	☒	A description of all covariates tested
☐	☒	A description of any assumptions or corrections, such as tests of normality and adjustment for multiple comparisons
☐	☒	A full description of the statistical parameters including central tendency (e.g. means) or other basic estimates (e.g. regression coefficient) AND variation (e.g. standard deviation) or associated estimates of uncertainty (e.g. confidence intervals)
☐	☒	For null hypothesis testing, the test statistic (e.g. *F*, *t*, *r*) with confidence intervals, effect sizes, degrees of freedom and *P* value noted<br>*Give P values as exact values whenever suitable.*
☒	☐	For Bayesian analysis, information on the choice of priors and Markov chain Monte Carlo settings
☒	☐	For hierarchical and complex designs, identification of the appropriate level for tests and full reporting of outcomes
☐	☒	Estimates of effect sizes (e.g. Cohen's *d*, Pearson's *r*), indicating how they were calculated

Our web collection on statistics for biologists contains articles on many of the points above.

Software and code

Policy information about availability of computer code

Data collection	jsPsych
Data analysis	python 3.5

For manuscripts utilizing custom algorithms or software that are central to the research but not yet described in published literature, software must be made available to editors and reviewers. We strongly encourage code deposition in a community repository (e.g. GitHub). See the Nature Portfolio guidelines for submitting code & software for further information.

Data

Policy information about availability of data

All manuscripts must include a data availability statement. This statement should provide the following information, where applicable:

- Accession codes, unique identifiers, or web links for publicly available datasets
- A description of any restrictions on data availability
- For clinical datasets or third party data, please ensure that the statement adheres to our policy

The dataset generated for this study is available in the Open Science Framework repository at https://osf.io/8xz3w/files/osfstorage
The code written for this study is openly accessible at: https://github.com/google-deepmind/hybrid_rnns_reward_learning/tree/main/hybrid_rnns_reward_learning

Research involving human participants, their data, or biological material

Policy information about studies with human participants or human data. See also policy information about sex, gender (identity/presentation), and sexual orientation and race, ethnicity and racism.

Reporting on sex and gender	Our analyses did not focus on gender and/or sex differences. Both males and females were recruited equally to participate in the study.
Reporting on race, ethnicity, or other socially relevant groupings	No reporting was done on race, ethnicity, or other socially relevant groupings.
Population characteristics	Our study did not focus on population characteristics or individual differences.
Recruitment	Participants were recruited using the standard approach of Prolific.com. The only restrictions were the limitation to adulthood (age >= 18), located in the UK, and mastery of the English language.
Ethics oversight	HuBREC, Google DeepMind's Human Behavioural Research Committee

Note that full information on the approval of the study protocol must also be provided in the manuscript.

Field-specific reporting

Please select the one below that is the best fit for your research. If you are not sure, read the appropriate sections before making your selection.

☐ Life sciences ☒ Behavioural & social sciences ☐ Ecological, evolutionary & environmental sciences

For a reference copy of the document with all sections, see nature.com/documents/nr-reporting-summary-flat.pdf

Behavioural & social sciences study design

All studies must disclose on these points even when the disclosure is negative.

Study description	We collected a large dataset from a reward-learning task in which human participants repeatedly chose among four possible actions, which were rewarded according to noisy reward magnitudes that drifted over time (a non-stationary 'bandit' task; Fig. 1E; Daw et al., 2006). On each trial of the task, participants selected one of the four actions and were given the corresponding reward. We collected a large dataset online (880 participants, 862 of whom passed inclusion criteria; 4,134 task blocks; 617,871 valid trials). The dataset is quantitative.
Research sample	We recruited 880 participants on Prolific (app.prolific.co). No statistical methods were used to pre-determine the sample size but our sample size is orders of magnitude larger than most traditional lab-based human experimental studies, and similar in size to those reported in previous publications focused on large-scale experiments. In agreement with the ethical guidelines of the Google DeepMind Human Behavioral Research Committee (HuBReC), all participants were local to the UK and fluent in English. Participants provided informed consent and were paid at a rate of 12 pounds per hour; there was no performance-based bonus payment. The study was not preregistered. Dedicated studies have shown that prolific samples rank high in terms of representativeness. Eighty participants were asked to complete one training and three testing blocks of 150 trials each. The remaining 800 participants were asked to complete one training block of 50 trials and five testing blocks of 150 trials, for a total of 4,240 task blocks. Four participants in the first (5%) and 14 participants (1.75%) in the second sample failed to finish the experiment and were excluded, leading to an initial sample of 880 - 18 = 862 participants who collectively finished (80 - 4) * 3 + (800 - 14) * 5 = 4,158 task blocks. We further excluded blocks in which participants missed more than 15 of the 150 trials (10%), 24 blocks in total (0.58%). Hence, our final dataset comprised 4,134 blocks (with 617,871 valid trials) from 862 participants. Of these 862 participants, 858 (99.5%) provided valid demographic information: 341 (39.7%) were female and 517 (60.3%) were male; the average age was 39.7 years, with a range of 18 - 88 and a standard deviation of 13.1 years.
Sampling strategy	Our sample size was chosen to be similar to the largest datasets in the literature concerning similar tasks. Our planned analysis methods (based on neural networks) required a larger dataset than typical in the field. Whereas most existing studies collect on the order of 30-50 data points, our study contains more than 4.000.
Data collection	Participants took the study on a computer in the privacy of their own homes, using the prolific platform. No researchers were present with research participants.
Timing	All participants were collected within approximately two days on 2023-04-20.
Data exclusions	Eighty participants were asked to complete one training and three testing blocks of 150 trials each. The remaining 800 participants were asked to complete one training block of 50 trials and five testing blocks of 150 trials, for a total for 4,240 task blocks. Four participants in the first (5\%) and 14 participants (1.75\%) in the second sample failed to finish the experiment and were excluded, leading to an initial dataset of $(80-4) \cdot 3+(800-14) \cdot 5=4,158$ task blocks. We further excluded blocks in which participants missed more than 15 of the 150 trials (10\%), 24 blocks in total (0.58\%). Hence, our final dataset comprised 4,134 blocks (comprising 617,871 valid trials) from 862 participants. Of these 862 participants, 858 (99.5\%) provided valid demographic information: 341

	(39.7\%) were female and 517 (60.3\%) were male; the average age was 39.7 years, with a range of 18-88 and a standard deviation of 13.1 years.
Non-participation	171 dropped out of the study. Reasons included running out of time (which was provided generously) and voluntarily dropping the task. Participants did not face negative consequences for opting out of the study.
Randomization	NA.

Reporting for specific materials, systems and methods

We require information from authors about some types of materials, experimental systems and methods used in many studies. Here, indicate whether each material, system or method listed is relevant to your study. If you are not sure if a list item applies to your research, read the appropriate section before selecting a response.

Materials & experimental systems

n/a	Involved in the study	
☒	☐	Antibodies
☒	☐	Eukaryotic cell lines
☒	☐	Palaeontology and archaeology
☒	☐	Animals and other organisms
☒	☐	Clinical data
☒	☐	Dual use research of concern
☒	☐	Plants

Methods

n/a	Involved in the study	
☒	☐	ChIP-seq
☒	☐	Flow cytometry
☒	☐	MRI-based neuroimaging

Plants

Seed stocks	NA
Novel plant genotypes	NA
Authentication	NA
